# The *Pectobacterium* pangenome, with a focus on *Pectobacterium brasiliense*, shows a robust core and extensive exchange of genes from a shared gene pool

**DOI:** 10.1186/s12864-021-07583-5

**Published:** 2021-04-14

**Authors:** Eef M. Jonkheer, Balázs Brankovics, Ilse M. Houwers, Jan M. van der Wolf, Peter J. M. Bonants, Robert A. M. Vreeburg, Robert Bollema, Jorn R. de Haan, Lidija Berke, Sandra Smit, Dick de Ridder, Theo A. J. van der Lee

**Affiliations:** 1 Bioinformatics Group, Wageningen University, Droevendaalsesteeg 1, 6708 PB Wageningen, The Netherlands; 2 Biointeractions and Plant Health, Wageningen Plant Research, Droevendaalsesteeg 1, 6708 PB Wageningen, The Netherlands; 3 Nederlandse Algemene Keuringsdienst voor zaaizaad en pootgoed van landbouwgewassen, Randweg 14, 8304 AS Emmeloord, The Netherlands; 4 Genetwister Technologies B.V, Nieuwe Kanaal 7b, 6709 PA Wageningen, The Netherlands

**Keywords:** *Pectobacterium*, Soft rot *Pectobacteriaceae*, Plant pathogen, Comparative genomics, Pangenome, Gene repertoire, Phylogeny, Virulence, *Pectobacterium brasiliense*

## Abstract

**Background:**

Bacterial plant pathogens of the *Pectobacterium* genus are responsible for a wide spectrum of diseases in plants, including important crops such as potato, tomato, lettuce, and banana. Investigation of the genetic diversity underlying virulence and host specificity can be performed at genome level by using a comprehensive comparative approach called pangenomics. A pangenomic approach, using newly developed functionalities in PanTools, was applied to analyze the complex phylogeny of the *Pectobacterium* genus. We specifically used the pangenome to investigate genetic differences between virulent and avirulent strains of *P. brasiliense*, a potato blackleg causing species dominantly present in Western Europe.

**Results:**

Here we generated a multilevel pangenome for *Pectobacterium*, comprising 197 strains across 19 species, including type strains, with a focus on *P. brasiliense*. The extensive phylogenetic analysis of the *Pectobacterium* genus showed robust distinct clades, with most detail provided by 452,388 parsimony-informative single-nucleotide polymorphisms identified in single-copy orthologs. The average *Pectobacterium* genome consists of 47% core genes, 1% unique genes, and 52% accessory genes. Using the pangenome, we zoomed in on differences between virulent and avirulent *P. brasiliense* strains and identified 86 genes associated to virulent strains. We found that the organization of genes is highly structured and linked with gene conservation, function, and transcriptional orientation.

**Conclusion:**

The pangenome analysis demonstrates that evolution in Pectobacteria is a highly dynamic process, including gene acquisitions partly in clusters, genome rearrangements, and loss of genes. *Pectobacterium* species are typically not characterized by a set of species-specific genes, but instead present themselves using new gene combinations from the shared gene pool. A multilevel pangenomic approach, fusing DNA, protein, biological function, taxonomic group, and phenotypes, facilitates studies in a flexible taxonomic context.

**Supplementary Information:**

The online version contains supplementary material available at 10.1186/s12864-021-07583-5.

## Background

Bacteria from the *Pectobacterium* genus (formerly *Erwinia*) are among the top ten economically most studied plant pathogenic bacteria, reflecting its economic importance [1]. They cause a broad spectrum of bacterial soft rot diseases (soft rot, blackleg, and stem wilt) in a wide host range of important crops [2, 3]. Considerable crop losses with high economic impact have been attributed to the bacterium on all continents [4, 5]. Pectobacteria differ from other soft rot bacteria by their large arsenal of pectinases that are used to degrade host tissue to acquire nutrients for bacterial growth [6, 7]. The *Pectobacterium* genus is a phylogenetically diverse group of Gram-negative, motile bacteria, belonging to the *Pectobacteriaceae* family [8]. In an era where the number of sequenced strains is rapidly growing, improved tools for comparative genomics are required to support phylogenetic and host pathogen research.

To assess genetic diversity and phylogeny of *Pectobacteria*, a comprehensive study involving isolates from different regions, different hosts and different species is required. Using next generation sequencing (NGS), the *Pectobacterium* taxonomy underwent major changes, resulting in nineteen described *Pectobacterium* species as of June 2020 [9]. The most recent additions to the taxonomy are *P. parvum* and the genomospecies *P. versatile*, which recently has been elevated to the species level [9, 10]*.* Correct species identification is difficult, and phylogenomic approaches that use the whole genome instead of a single phylogenetic marker prevent misclassification [11, 12]. The improved comparative methodologies also allow to correctly diagnose a number of strains in culture collections that were previously misclassified.

One of the most important Pectobacterium species nowadays is *P. brasiliense*. *P. brasiliense* was initially described as an atypical *Erwinia carotovora* strain causing blackleg on potato tubers in Brazil [13], and was invalidly classified to be a subspecies of *P. carotovorum* [14]*.* Recently, it was elevated to the species level based on whole genome sequence analysis [10]. Only a few years after its identification, the species emerged as a global problem, with many reports that indicate a broad range of plant hosts associated with soft rot symptoms in locations across the world [15–18]. After *P. brasiliense* was first discovered in Belgium in 2012, it quickly became a dominant blackleg causing pathogen on the European continent [16, 19]*.* However, several comparative field studies showed high phenotypic variation in the virulence among *P. brasiliense* isolates [13, 20, 21].

The comparison of genomes provides insights in genetic mechanisms, evolution, and the translation of genotypes into phenotypes [22]. Traditionally, genomes are compared pairwise, or centered on a single reference. However, advances in next-generation sequencing technologies (NGS) have made the reconstruction of genomes easier and more accessible. Computationally, pairwise methods fail to scale to the large number of genomes currently available. Moreover, a single reference genome cannot account for the intraspecific variability found in nature. To reflect the notion of bacterial species more accurately, the concept of a pangenome was introduced [23]. The pangenome is an abstract representation of the genomes of all the strains that are present in the population, species or genus.

In recent years, efficient methods to construct sequence-level pangenomes were reported [24–27], as well as tools for gene-level pangenome analyses [28–30]. However, the integration of whole genomes and functional information for biological analysis in a scalable platform, not limited to prokaryotes, remains challenging. Our pangenomic analysis platform PanTools [31] has a hierarchical data structure, including sequence data (represented as a localized, compressed De Bruijn graph), structural/functional annotations, and crosslinks between DNA and protein sequences and annotations. In addition to pangenome-graph construction, PanTools includes a method for de novo detection of homology groups that contain both orthologous and paralogous sequences.

Aims of this study were (i) to extend the functionality of PanTools inducing phylogenetic and phenotypic modules as well as a variety of downstream analysis methods, (ii) to exploit the genomic information in the *Pectobacterium* genus, by constructing a comprehensive pangenome of 197 genomes with a focus on *P. brasiliense* for which we isolated and sequenced a large set of additional isolates, and (iii) within this phylogenetic context we classified the gene repertoire into core, accessory, unique, or specific to a certain clade or function or GO-term. Finally (iv) field bioassays were performed to assess the virulence of a set of 40 *P. brasiliense* isolates on different potato varieties and we identified genes that are associated to the virulent phenotype. Our pangenome study underlines the benefits of such an integrated approach to understand genome function and evolution of complex plant pathogens.

## Results

### A novel collection of high-quality *Pectobacterium* genomes capturing genetic diversity

To capture the genetic diversity in the genus *Pectobacterium* and to extend the limited collection of available genomes, we sequenced 63 Pectobacterium isolates (55 *P. brasiliense*, 3 *P. versatile*, 1 *P. aquaticum*, 1 *P. parmentieri,* 2 *P. polaris* and 1 *P. punjabense*) de novo using both Illumina and PacBio technologies (Additional file 1: Table S1). The resulting genome assemblies varied in size (4.3 to 5.3 Mbp), GC content (50.29 and 52.17%), and fragmentation (1 to 601 contigs per genome, with a median of 49; 19 strains were assembled into a single contig). All genomes were annotated with Prokka [32], which resulted in 3944 to 4719 predicted protein-coding genes and 35 to 84 tRNA genes per genome. The number of predicted genes per genome strongly correlated to the genome size (Pearson’s correlation coefficient of 0.88). Only minor differences were found in the number of functional annotations per strain, including GO terms, Pfam domains, and biosynthetic gene clusters.

This set of novel genomes was combined with 150 publicly available *Pectobacterium* genomes from Genbank, resulting in a grand total of 213 strains (Table S1). As comparative genomic approaches highly depend on genome quality, we estimated genome completeness using the BUSCO v3 data set *Enterobacteriales* odb9 (781 orthologs) [33]. The scores ranged from 73.6 to 99.7% with a median of 99.6%, indicating general near completeness of the genomes and reliable protein annotation. Interestingly, several genes were missing, fragmented or duplicated in up to 97.5% of all strains, indicating that these genes are not truly universal in the genus *Pectobacterium.* Aiming to compare high-quality genomes only, we excluded all genomes with a BUSCO score below 99%, resulting in a final set of 197 high-quality genomes.

Subsequently, verification or assignment of the correct species to each genome was performed using the average nucleotide identity (ANI) score, a widely accepted genome-based method, in combination with the type strains to verify or correct the species identification. The threshold for assigning a genome to a species was a minimum of 95% identity to the type strain of that species [34]. Of the publicly available data, a species name could be assigned to 9 unclassified genomes while in 16 cases the species name was corrected (Additional file 1: Table S2).

The lowest ANI score observed between genomes was 82.8%, confirming all genomes represent a single genus given the threshold ANI score of 75.0% or higher, usually applied for the genus level (Additional file 1: Table S3) [35]. Lowest ANI scores between members of the same species were all above 95%, except for *P. brasiliense*, with the lowest being 93.9%. More than three quarters (70 out of 92) of the low scores between *P. brasiliense* members were caused by strain IPO 0590, with an ANI score of 95.2% to the type strain (LMG 21371). The remaining low ANI scores were found in comparisons against strains NAK 468, NAK 470 and NAK 433. After *P. brasiliense,* the lowest ANI distances observed were in the species *P. aquaticum* 95.7% and *P. polaris* 96.8%. The highest ANI scores were found for species for *P. parmentieri* (98.8%), *P. atrosepticum* (98.8%), *P. odoriferum* (98.6%) and *P. versatile* (97.6%). Finally, one strain (NAK 253) did not fall within any of the species, indicating that this could be a new species.

### A *Pectobacterium* pangenome across multiple species

The collection of 197 high-quality *Pectobacterium* genomes across 19 species served as input for construction of the pangenome using PanTools v3. First, the genome sequences were split into k-mer subsequences and compressed into a De Bruijn graph. Next, the genome annotations (mRNAs and their encoded proteins) were added to the pangenome. Finally, phenotypic data, such as species and virulence, were coupled to each strain.

Fundamental to a pangenome analysis is determining the phylogenetic relationships between homologous sequences in all strains. Correctly inferring homology is complex due to unknown evolutionary distances and dynamic remodeling of genome content through gain, loss, duplication and transfer of genes. Therefore, we developed a novel strategy using BUSCO’s universal single-copy orthologs to select the optimal settings for homology grouping in PanTools. We performed the grouping using eight different settings, ranging from strict to relaxed, and applied BUSCO benchmarking to assess which setting results in clusters that agree most with 670 BUSCO genes (Table 1). This benchmark showed a clear trade-off between recall, which reflects the method’s ability to cluster true homologs together, and precision, showing the ability to separate non-homologs. For this pangenome, the D4 setting (minimum sequence similarity of 65%) was optimal with the highest F-score (99.98%) and the largest number of correct groups (640), grouping all proteins into 22,347 homology groups of which 1699 were single-copy (1 to 1) orthology groups.

**Table 1:** General and BUSCO benchmark statistics for homology grouping performed under setting D1 to D8

Clustering setting	Minimum sequence similarity	Homology groups	Single copy groups	Correct groups ^a^	True Positives	False Positives	False Negatives	Recall	Precision	F-score
D1	95%	49,290	812	395	128,085	14	3905	0.9704	0.9999	0.9849
D2	85%	28,896	1615	629	131,795	24	195	0.9985	0.9998	0.9992
D3	75%	24,650	1690	638	131,952	35	38	0.9997	0.9997	0.9997
D4	65%	22,347	1699	640	131,975	38	15	0.9999	0.9997	0.9998
D5	55%	20,636	1683	639	131,975	44	15	0.9999	0.9997	0.9998
D6	45%	19,234	1653	633	131,985	245	5	0.9981	0.9981	0.9991
D7	35%	17,908	1612	623	131,985	508	5	0.9962	0.9962	0.9981
D8	25%	16,486	1486	607	131,986	7002	4	1.0000	0.9496	0.9741

^a^ Correct groups are defined as the number of groups that correctly organize one out of 670 ‘complete’ and ‘non-duplicated’ *Enterobacteriaceae* BUSCO genes. Calculations of recall, precision, and F-score explained in Methods

### Pangenome characterization

**Fig. 1:**
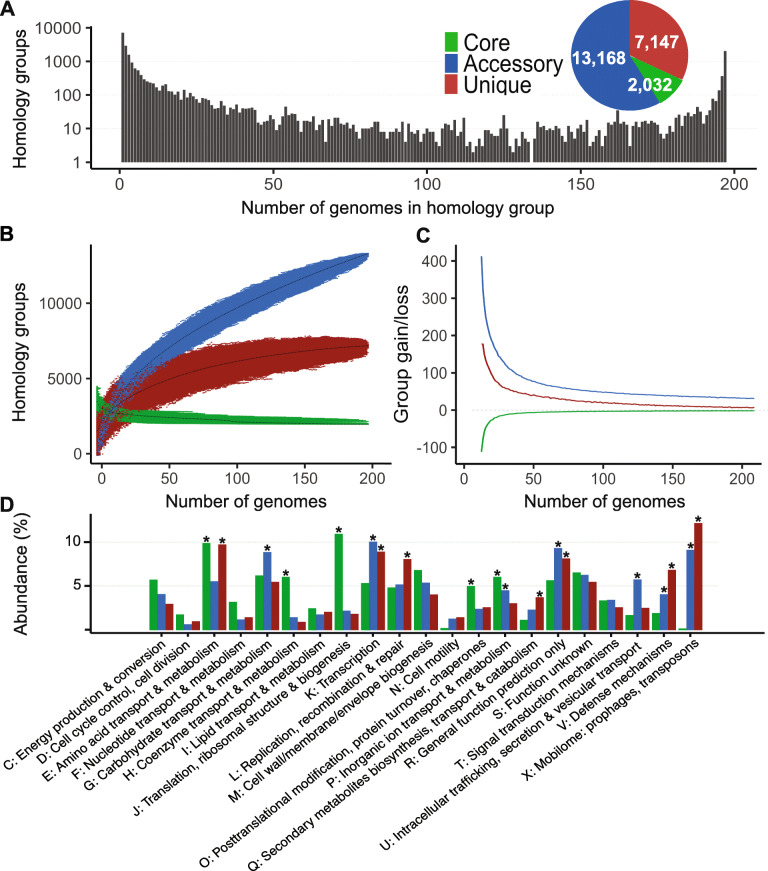
The *Pectobacterium* pangenome. **a** The distribution of sizes of all (22,347) homology groups. The size of a homology group is determined by the number of distinct genomes. **b** Simulation of increase of the pangenome size and the decrease of core-genome size on gene level using random sampling of genomes over 10,000 iterations. Colored dots represent the number of classified homology groups for random genome combinations, black dots indicate the median of each category. **c** Average gene gain and loss by the addition of genomes to the pangenome. **d** Proportion of COG functional categories assigned to homology groups. The asterisk above bars denotes the COG category is significantly (*p* < 0.05) more abundant in that class than other gene classes (core, accessory, unique). COG categories A, B, W, Y, Z were excluded considering their abundance below 1%

The gene repertoire in a pangenome can be separated into core genes (present in all genomes), accessory genes (present in some genomes, but not all), and unique genes (present in a single strain). This labeling is done at the level of homology groups, which shows the exact occurrence of each gene in all constituent genomes. In the *Pectobacterium* pangenome, we classified 2032 (9.1%) groups as core, 13,168 (58.9%) as accessory and 7147 (32.0%) as unique. An average *Pectobacterium* genome consists of 46.9% (σ 1.9) core genes, 0.9% (σ 1.3) unique genes, and 52.3% (σ 2.6) accessory genes (Additional file 2: Figure S1). The distribution of homology group sizes (Fig. 1a) shows that the majority of genes were either conserved across the entire genus or specific to one or few genomes. This is confirmed by calculating that 2853 groups (12.8%) belong to the so-called softcore (containing genes from more than 95% of the genomes), and 10,077 groups (45.1%) can be classified as ‘cloud’ (represented by less than 1% of the genomes) (Additional file 2: Figure S2).

We tested the impact of annotation mistakes on the core genes by BLASTing the genes of 364 homology groups that were present in all but one genome, against the genome where the gene was not found. For 20 groups (5.5%) the gene was found fully in the genome sequence but was missed by the gene annotation. The validity of unique genes was assessed by taking genes present in a single genome that lack a function or domain and BLASTing these against NCBI’s nr database. Over 90% of the total unique homology groups had an assigned function or significant BLAST hit (E-value < 1.0e-5). Based on these findings the effect of the annotation quality on our analysis was shown to be minimal and loosening the thresholds for calling genes core and unique was not deemed necessary.

The comprehensiveness of the available information in the pangenome is assessed by noting the shifts in the number of core, accessory, and unique groups upon the addition of genomes to the pangenome. Figure 1b illustrates that core and unique groups have nearly stabilized and reached a plateau, while the number of accessory groups still increases. Figure 1c shows the average group gain and loss caused by increasing the number genomes. The core genome stabilized after approximately 15 genomes, although it slightly decreased for every genome added. This pattern was similar for the identification of unique genes. The high increase in the number of accessory groups slowed quickly, but the gain is still significant even near the full pangenome size. We fitted Heaps’ law [36] to the number of newly discovered unique genes per additional genome which resulted in a decay rate (α) of 0.53. According to Heaps’ Law, when *α* < 1, the pangenome can be considered open, thus the *α* of 0.53 indicates the Pectobacterium pangenome is open. Adding a final genome to a pangenome of the remaining 196 on average leads to an increase of 6.5 (σ 50.9) unique and 29.8 (σ 35.1) accessory groups, with a loss of 1.9 (σ 13.9) core groups. Nearly half (87) of the strains were *P. brasiliense*. To assess the impact of such a large subsample on the openness of the pangenome, we estimated the size of the *P. brasiliense* pangenome and of the remaining *Pectobacterium* spp. separately. Both pangenomes showed a similar open pangenome structure which were supported by a Heaps’ law decay rate below 1. The pangenome of 18 *Pectobacterium* spp. had a decay rate of 0.69, but more interestingly, the *P. brasiliense* pangenome gained more new genes per additional genome and had a lower decay rate of 0.51 (Additional file 2: Figures S3-S6).

To enable the biological interpretation of the various homology groups, we integrated information found in the Gene Ontology (GO), InterPro and Pfam databases into the pangenome. At least one type of functional annotation was assigned to 16,073 homology groups: 99.9% of the core, 74.4% of the accessory and 59.5% of the unique groups. Furthermore, we compared the protein sequences in the pangenome to the Clusters of Orthologous Groups (COG) database to which one third of the proteins had a significant hit (> 65% identity, E-value < 1.0e-5). The percentage abundance of COG categories was used to identify differences between the core, accessory and unique protein sets (Fig. 1d). As expected, the core was enriched for housekeeping COG functions: Nucleotide transport and metabolism (COG category F), Coenzyme transport and metabolism (H), and Translation, ribosomal structure and biogenesis (J). Interestingly, Amino acid transport and metabolism (E) was high in abundance for core and unique groups, but low for accessory groups. Overrepresented functions for accessory genes were: Transcription (K), Secondary metabolites biosynthesis, transport and catabolism (Q), Defense mechanisms (V), and Mobilome: prophages, transposons (X).

### Phylogenomics based on core genome single-nucleotide-polymorphisms

**Fig. 2:**
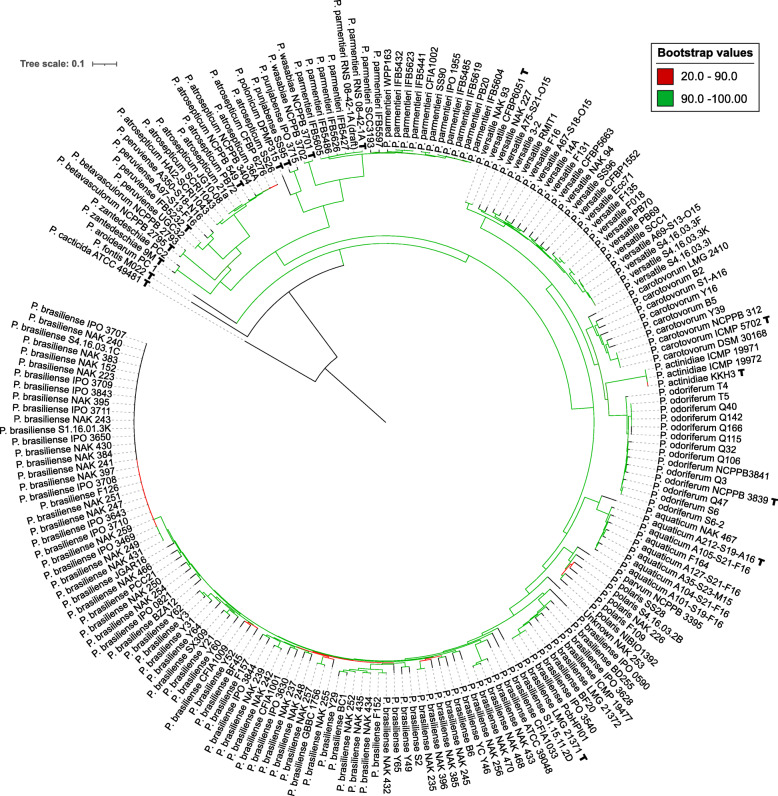
Maximum likelihood core SNP phylogenetic tree. The tree was inferred on a total of 452,388 SNPs extracted from the alignments of 1699 single copy orthologous genes. The tree was rooted using *P. cacticida* (ATCC 49481) as outgroup. Branches are colored according to their bootstrap support value obtained from 1000 bootstrap replications

To understand the relationships of sequenced strains within the *Pectobacterium* genus, a phylogenetic tree was constructed from 452,388 parsimony informative sites of single-copy orthologous genes (1699 in total) present in all genomes. The inferred maximum likelihood (ML) phylogeny is presented in Fig. 2, a rectangular version of the phylogeny was added as Additional file 2: Figure S8. All species clustered in a separate clade, including the newly characterized *P. aquaticum*, *P. versatile* and *P. parvum*. Strain NAK 253, which could not be assigned to a species, was placed between the *P. brasiliense*, *P. polaris and P. parvum* species. In addition, some clades within the *P. brasiliense* species were identified. High bootstrap (> 90) support was found for all species clades. Only one subclade of 33 genetically closely related *P. brasiliense* strains had low bootstrap support due to the very limited number of single nucleotide polymorphisms (SNPs) that differentiate these strains. Strain *P. cacticida (*ATCC 49481*)* was most distant to all strains based on shared gene content and was therefore selected as outgroup to root the tree. To validate the outgroup, the core SNP method was applied to a separate pangenome with *Pectobacterium* and *Dickeya* strains (*D. dadantii* 3937, *D. paradisiaca* Ech703 and *D. zeae* Ech586) which confirmed the phylogenetic position of *P. cacticida*(Additional file 2: Figure S6).

### A robust phylogeny using distinct methods

From an initial set of 17 housekeeping genes commonly used for multilocus sequence analysis (MLSA) [16, 37–39], we selected five genes that were present in single copy in all genomes and showed the highest genetic diversity: *acnA*, *dnaX*, *gyrA*, *gyrB* and *mtlD* (Additional file 1: Table S4). The commonly used marker gene *gapA* [40] was not included since each of our selected genes had a 3-to-9-fold higher number of SNPs. The inferred phylogeny (based on a total of 2996 informative sites) showed that this method could accurately separate all species into distinct clades, using only 5 genes out of 1699 single copy orthologs (Additional file 2: Figure S9). However, bootstrap values between 40 and 60 indicated moderate to high levels of uncertainty within the *P. parmentieri*, *P. aquaticum* and *P. brasiliense* clades. The placement of the *P. fontis* (M022) and *P. aroidearum* (PC1) genomes was likewise ambiguous due to insufficient bootstrap support (value of 59).

In addition to the implemented ML methods, Neighbor-Joining (NJ) based methods were applied that use the features stored in the pangenome graph database. First, we converted the scores from the ANI species identification analysis into distance values (d = 1-ANI), from which a tree was inferred that accurately distinguished all species (Additional file 2: S10). For our second NJ tree we exploited the De Bruijn graph data structure of the pangenome to calculate the distance between two genomes based on shared k-mers. This alignment-free method ignored the genome structure and only considered the absence or presence of 17 bp k-mer sequences. The k-mer based phylogeny was congruent to the core SNP tree (Additional file 2: Figure S11).

Our final phylogeny was inferred from gene distances based on the shared number of genes that were identified through the homology groups. Nearly all strains grouped according to their species, except for six *P. brasiliense* strains that were placed distantly in two distinct clades. (Additional file 2: Figure S12). The first clade, represented by strains NAK 433, NAK 468, NAK 470, was closely related with short branch lengths and ANI scores of 99.0–99.6%. These three genomes shared between 81.4 and 94.6% of their gene content while they had respectively, 53, 5 and 21 unique homology groups. In contrast, the second clade, represented by strains CFIA1033, IPO 0590, and S1.15.11.2D, showed high variability with long branch lengths in the phylogeny and ANI scores of 94.8–96.1%. Comparison of the three genomes showed they had little in common; only 67.4 to 71.2% of their gene content was shared and genomes had a relatively high number of unique genes (102, 207, and 106, respectively).

We compared the phylogenies resulting from the different methods in terms of accordance and resolution. Four out of the five methods (ANI, MLSA, k-mer, core SNP tree) were able to distinguish all species in separate clades. For the method using gene content this was possible for 97% of the genomes, as described above. The resolution as observed from the branch lengths and bootstrap values varied for the different methods, with the core SNP phylogeny providing the highest resolution. For the four phylogenies that correctly clustered the species we observed some incongruities inside certain clades, mostly located at the base of the *P. brasiliense* clade. To estimate the concordance of the tree topologies, the IQ-tree AU-test was applied two consecutive times to calculate the log-likelihood of trees based on the alignment and model parameters of an ML tree. First, with the MLSA phylogeny as reference, the gene content tree was rejected based on the estimated parameters of the ML tree. Using the SNP tree as reference model, only the k-mer distance phylogeny was within the confidence limit of the selected topology.

**Fig. 3:**
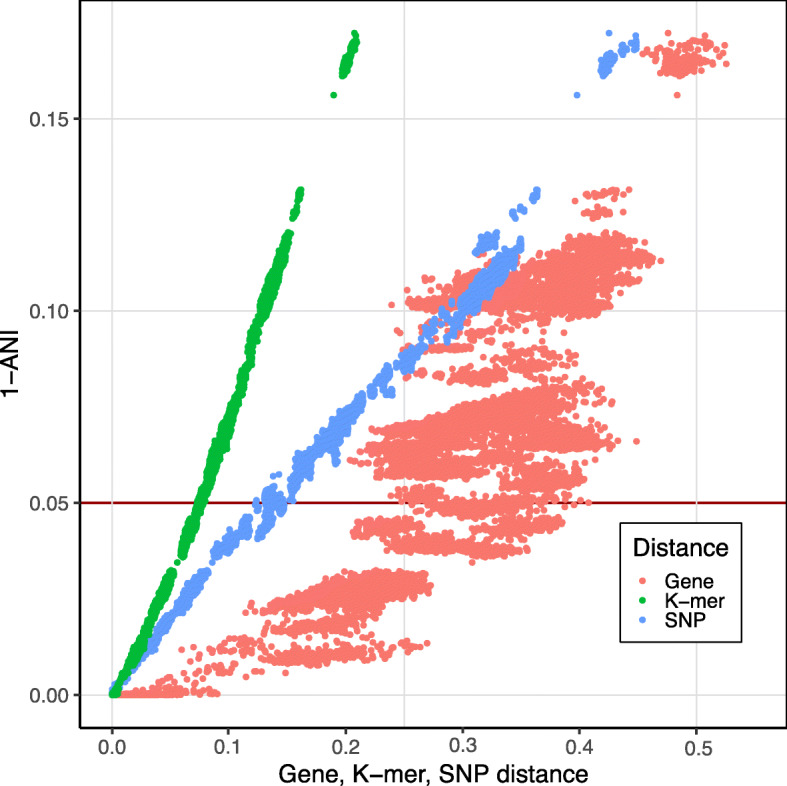
Difference in phylogenetic distances. The ANI score between two genomes is plotted against the calculated SNP (blue), gene (red) and k-mer (green) pairwise distances. The red horizontal line highlights an ANI score of 95.0, a frequently used threshold for species delineation

To further examine how robust phylogenetic comparisons are, we plotted the pairwise distances found by the core SNP, k-mer, and gene content methods against the ANI distance (Fig. 3). Distances from the MLSA were excluded, as this method is in fact a low-resolution version of the core SNP tree based on a preselection of genes. The plot revealed a strong linear relationship between the ANI, SNP and k-mer distances (Additional file 1: Table S5). In sharp contrast, the genome content showed a relatively wide variation at nearly any distance. Moreover, the plot reveals that two highly similar *Pectobacterium* strains with an ANI score over 99% can vary from 5% up to 25% in gene content whereas two strains at the species boundary (ANI ~ 95.0%) vary between 25 and 40%.

### Discovery of species-specific genes

To study evolution of a trait, speciation or niche/host adaptation it is of interest to identify genes that are specific for a species or phylogenetic branch of the *Pectobacterium* pangenome. We used our comprehensive set of genomes together with ANI verified species names for identification of such species-specific genes. We excluded species with less than 5 genomes from this analysis, leaving eight species which were subsequently assessed. We identified 9 species-specific homology groups for *P. aquaticum*, 52 for *P. atrosepticum*, 53 for *P. parmentieri,* 46 for *P. odoriferum* and a single group was specific to *P. carotovorum* (Additional file 1: Table S6, S7). No species-specific homology groups were identified for the two best sampled species, *P. brasiliense* (87 genomes) and *P. versatile* (24 genomes). Furthermore, the analysis indicates that the identification of species-specific genes or homology groups largely depends on the genetic diversity of the sequenced isolates and not so much on the number of isolates. This is exemplified by *P. polaris*, which was represented by only five genomes that originate from different geographic locations across the world: Morocco, Canada, Russia and Pakistan. These five strains displayed high genetic diversity (reflected in an ANI score of 95.9–97.0%, shared gene content between 73.7 and 80.1% and high SNP distance) and no species-specific genes could be identified.

### Tracing virulence in *P. brasiliense*

We searched for genetic differences between genomes of virulent and avirulent strains using our pangenome approach. Virulence was assessed by field tests and phenotypic assays in two consecutive years for a selection of *P. brasiliense, P. punjabense*, and *P. aquaticum* strains sampled across the Netherlands (Additional file 1: Table S1). For some of the publicly available strains virulence information was reported; however, this information was not included as the bioassays performed were very different. Fifteen *P. brasiliense* strains were assessed as virulent, while 25 showed either marginal or no virulence. The virulent strains were highly similar (ANI ≥ 99.93%) and shared at least 94.1% of their genes. In contrast, avirulent strains did not cluster together in the phylogeny; their ANI scores ranged from 95.5 to 99.9% and they shared at least 63.2% of their gene content.

We searched the pangenome for candidate genes associated to virulence based on their presence in virulent strains and their absence in avirulent ones. For this analysis, strains without pathogenicity data were ignored. No virulence-specific genes were initially identified. Comparing the virulence phenotype and the phylogeny of the strains revealed that two strains (NAK 223 and NAK 259) that appeared avirulent in the field tests clustered inside the virulent group. Possibly, the pathogenicity of these two strains was affected by secondary mutations. When we ignored these two genomes, 86 homology groups were identified that were present in all virulent strains and absent in the avirulent strains. Vice versa, 12 homology groups were found absent in all virulent strains and present in the avirulent ones (Additional file 1: Table S7). There were 86–88 virulence-specific genes per genome as some genomes had additional copies of specific groups. Most genes (66 out of 86) were co-localized, i.e. had at least one neighbor that was also virulence specific. The gene order of these co-localized genes was conserved among all virulent strains: two gene clusters of six genes, four clusters each of four and three genes and 13 gene pairs (Additional file 1: Table S8). Functional annotations connected to the identified genes, included a Lysozyme inhibitor, Toll/interleukin-1 receptor, ABC-type siderophore export system, as well as multiple effector proteins and nonribosomal peptide synthetases. Statistically significant enrichment was observed for GO terms related to recombination and DNA modification with, in particular DNA methylation (Additional file 3). In addition, the virulent strains have four additional Pfam protein domains not present in any of the avirulent strains.

As we were unable to differentiate the two avirulent strains (NAK 223 and NAK 259) from virulent strains based on gene content, we aligned the sequences of single copy groups from the 15 highly similar (ANI ≥ 99.93) genomes to identify non-synonymous mutations. A total of 4237 single-copy groups were determined, which represents more than 95% of the individual gene content of a strain. Only inside the chaperone protein *dnaK*, a lysine was substituted by asparagine on position 92 in one avirulent and two virulent strains; however, a single variant that could discriminate the two avirulent strains from the virulent group could not be identified.

**Fig. 4:**
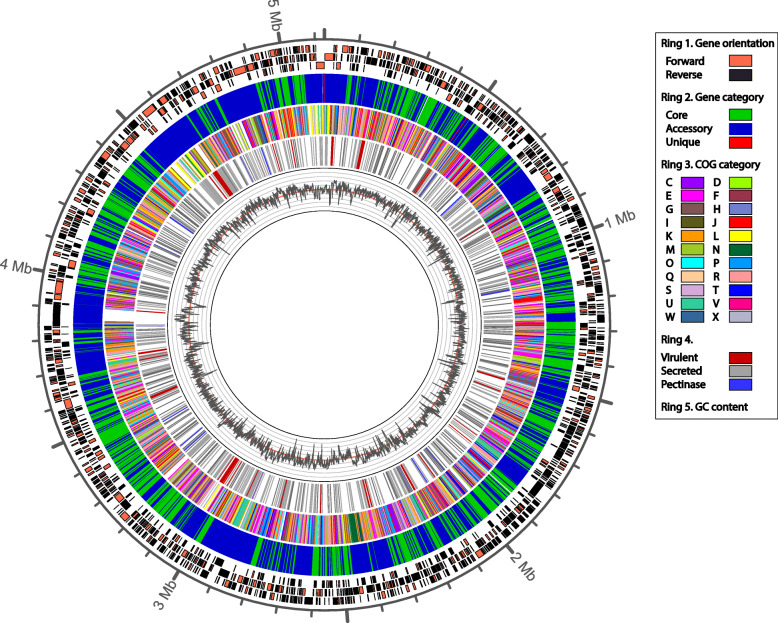
Circular chromosome map with genomic features of *P. brasiliense* NAK 240. Ring 1 displays three rows with blocks of neighboring genes in the same orientation, forward (black) or reverse (orange). Ring 2 holds genes colored by their classification: core (green), accessory (blue) or unique (red). Genes in the third ring are colored by COG category, genes without a significant hit to the COG database are white. Ring 4 displays 88 genes (86 groups) specific to virulent strains (red), secreted proteins identified by Phobius (grey), and pectinases (blue): *pelA*, *pelL*, *pelW*, *pelX*, *pelZ*, *pel1*, *pel2*, *pel3*, *pemA*, *pemB*, *ogl*, *rhiE*, *ganB* and *pnl*. Ring 5 shows GC content within the range of 25 to 75% (red line at 50%). Percentage of GC was calculated for blocks of 1000 bp

A circular genome plot was created to visualize genome organization and to incorporate the identified genes using strain NAK 240 as a validated and representative example for all virulent *P. brasiliense* strains (Fig. 4). Previous results of the gene classification, association of COG function and genome characteristics such as GC content and gene orientation were integrated into the overview. The figure shows how nearly all virulent genes seem to coincide with negative skews in the GC content. To support this observation, we compared the GC ratio of the virulence-specific genes to the rest of the genome; 81 of the 88 genes were below the average GC content (52.1%) of protein coding sequences in strain NAK 240. Finally, virulence was assessed by identifying pectolytic enzymes in the pangenome, based on the study of Li et al., (2018) [41] and Duprey et al., (2019) [42]. Out of the range assessed, ten pectin degradation genes (*ogl*, *pehX*, *pehA*, *pemA*, *pel1*, *pel3*, *pelX*, *pelW*, *pelA*, *pelL*) were found in all 197 genomes, some had duplicated copies in nearly all genomes (Fig. 4; Additional file 2: S7).

## Discussion

Pangenomes are becoming widely used to represent, analyze and predict the genomic diversity for large populations of a single species or genus. In this study we have integrated phylogenetic tools, the possibility to add functional and phenotypic annotations as well as quality control and selection procedures in PanTools to perform such pangenome analyses effectively. We applied these new functionalities on the complex genus *Pectobacterium* to build a comprehensive phylogeny that can guide *Pectobacterium* research and identified genes and mutations that are specific for clades or phenotypes that could be used as diagnostic markers.

### Quality assurance for a reliable pangenome

To ensure the quality of data analysis, we checked genomes before inclusion in the pangenome and optimized the clustering settings to assign proteins to the correct homology group. We used BUSCO [33] to verify completeness and used genomes that had a BUSCO score of at least 99%. The highest score was 99.7% and the median score was 99.6% of the original set. A closer analysis of the BUSCO output revealed that some genes were not found in specific lineages of the *Pectobacterium* set; removing these genes from the dataset increased the median score to 99.9%.

Classifying individual genes into homology groups is a crucial step in pangenomic analysis. The composition of homology groups is affected by several parameters, of which protein similarity cut-off is the most influential. The appropriate setting depends on the genomic diversity of included genome sequences. For the genus *Pectobacterium* we applied a new strategy for verification of the settings using a BUSCO reference set based on orthologs from OrthoDB [43]. As BUSCO genes should cluster separately in single-copy orthology groups, we found the optimal grouping was obtained in the *Pectobacterium* pangenome using a 65% similarity cut-off, yielding recall and precision scores of 99.9%. These results were further corroborated by the fact that the 2032 core homology groups in *Pectobacterium* are highly enriched in functions that relate to the maintenance of basal cellular functions. The size of the core genome is much smaller compared to the largest *Pectobacterium* pangenome found to date, which was estimated to contain 3171 core genes [41]; however, their study included less species (7 instead of 19) and less genomes (84 instead of 197).

A pangenomic approach relies on correct homology grouping. Care should be taken in the interpretation of gene classification, as both the core and unique parts of the pangenome are very sensitive to low genome and annotation quality. By applying rigid quality control settings and validating the correctness of core and unique homology groups, we could set strict core and unique cut-offs where in other studies, to circumvent the impact of genome and annotation quality, thresholds are loosened to allow for discovery of core and unique genes [44, 45]. Furthermore, we examined the exceptional cases in which genes appear to be absent or present in only one of the 197 genomes. Based on BLAST searches we demonstrate that genes can indeed be absent in a single strain and were not missed due to misannotation, while unique genes are likely to be true genes as over 90% share significant homology with genes outside the *Pectobacterium* genus*.* As the size of pangenomes will only expand in the future, quality assurance of the data will gain further importance.

### Effect of an uneven distribution of genetic diversity

Due to the large and diverse collection of *Pectobacterium* genomes presented in this study, the size and openness of the pangenome can be assessed. The *Pectobacterium* pangenome of 197 strains comprising 19 species seems to be closing, because on average only 6.5 (σ 50.9) new genes were gained on the last added genome in the pangenome size estimation. Remarkably, the strongly represented *P. brasiliense* subset of 87 genomes still gained more new genes on the last added genome compared to the pangenome with 110 genomes from 18 different species. Thus, although nearly half of the strains belong to the same species, relatively more novel genes are likely obtained by adding more *P. brasiliense* genomes to the pangenome. This correlates with the genomic diversity of *P. brasiliense* being the highest of all *Pectobacterium* species. The lowest ANI score within *P. brasiliense* is > 93.9%, followed by > 95.7% in *P. aquaticum* and > 96.8% in *P. polaris*. Accordingly, these three species also have the highest gene distance between species members: 41.3, 28.9, and 26.3%, respectively. In contrast, most other species show a much higher genetic similarity with an ANI score close to 99%: *P. atrosepticum* > 98.8%, *P. odoriferum* > 98.6% and *P. parmentieri* > 98.8%. The *Pectobacterium* pangenome does not have an infinite gene pool but given the Heaps’ law decay rate (0.53) and an uneven distribution of genomic diversity it should not be considered closed, which is in line with the open pangenome structure observed in other bacteria [41, 46]. For some species or clades within species such as the virulent *P. brasiliense* accessions (ANI ≥ 99.96), the available diversity may be largely covered in the current *Pectobacterium* pangenome and these parts can be considered saturated.

### Phylogeny relationships revealed in the *Pectobacterium* pangenome

Phylogenetic reconstruction is an essential part of all comparative genomic approaches. In our pangenomic analysis we applied and compared five different commonly used tree-reconstruction approaches: ANI, MLSA, a SNP tree derived from single-copy groups, k-mer and gene content. The five methods are distinct in strategy, exploiting alignment-based methods, known genes or the complete genomic content. Despite these differences, results were found to be largely in accordance and represented the taxonomic relationships accurately.

Of the five constructed (phylogenetic) trees, a phylogenomic approach based on the SNPs from all single copy orthologous genes had the highest resolution. This core SNP tree provides an accurate representation of evolutionary relationships within the *Pectobacterium* genus and was congruent to the phylogeny reported by Pasanen et al. (2020) [9]. The core SNP tree topology was compared using the AU-test [47, 48], which rejected similarity to all other phylogenies except for the k-mer distance tree. Given that the core SNP tree was inferred from 452,388 SNPs and only a few branches within *P. brasiliense* are ambiguous*,* the confidence interval for the AU-test was likely to be narrow, as only minor differences were found with the ANI and MLSA tree topologies. Considering that the number of genomes in a pangenome will continue to grow, the SNP method has a clear downside, which is the runtime. For aligning sequences and the ML inference we used MAFFT [49] and IQ-tree [47], respectively, two highly efficient tools that handle large datasets and scale accordingly to the number of genomes. However, when a pangenome contains thousands of genomes, this alignment-based method will eventually become too time consuming. Therefore, the k-mer based method offers a good alternative, since it is computationally the most efficient and showed the highest match to our core SNP phylogeny. Similarly, many alignment-free techniques were developed to address such issues with scalability [50, 51].

Species-level identification is required for the discovery of species-specific genes; however, incorrect species names in public datasets are a common phenomenon [52, 53]. Therefore, we used an ANI score of ≥95% with respect to a type strain for classifying strains into species [35, 54, 55]. In this way, we could prevent inconsistencies in the downstream analysis. Only one strain, NAK 253, could not be classified into any of the known *Pectobacterium* species. We hypothesize that this could be a new species most related to *P. polaris, P. brasiliense* and *P. parvum*. Our analysis also indicates a high genetic diversity among *P. brasiliense.* Genomes of *P. brasiliense* consistently grouped in three to four distinct clades in the k-mer and core SNP trees and may represent subspecies. Another interesting strain was NAK 467, identified as *P. aquaticum,* isolated in the Netherlands from a side channel of the river Meuse that springs in France. So far *P. aquaticum* was only reported in France [56], where it was found to spread via river waters.

### Species-specific genes, search for a ghost?

One of the initial intents of this study was to exploit the pangenome to identify species-specific genes and larger regions of colocalized genes. Previous attempts to identify these were often confounded by the diversity found when more ecological niches or geographic regions were sampled. We envisioned that a comprehensive pangenome approach would eliminate this pitfall and would allow for a better selection of genes. As the vast majority of proteins and homology groups are present only in certain genomes in the pangenome and accessory genes represent the highest percentage in each individual genome, there are numerous candidates that could be specific for a species. However, our study demonstrates that for a particular species, such as *P. brasiliense,* none of these accessory genes is specific for a species. As we verified that the correct identification at the species and sub-species level by ANI and all phylogenies is consistent with the correct classification of strains to species, we conclude that for the best-sampled species in the *Pectobacterium* pangenome, *P. brasiliense,* species-specific genes do simply not exist. Similar results were found for *P. carotovorum*, *P. versatile*, and *P. polaris.* For the species *P. aquaticum*, *P. atrosepticum*, *P. parmentieri* and *P. odoriferum* several candidate species-specific genes persisted, we hypothesize that increasing the number of genomes will further reduce this number and that species-specific genes are essentially not a constructive concept for *Pectobacterium*.

Horizonal gene transfer (HGT) is one of the main mechanisms in prokaryotic evolution for the lateral exchange of genes. That *Pectobacterium* species can adapt to environmental changes through HGT events has been observed in several studies [57–59]. Although the frequency of recombination events is hypothesized to significantly drop below ANI 95% [34, 60], these events are still likely to occur and exchange genes throughout a population. The notion that a shared gene pool, fostering new combinations of homology groups, drives evolution, further emphasizes the need for a pangenomic approach. In addition, loss of genes seems an important driver of evolution: it is common even in genetically closely related isolates (ANI > 99%) [61, 62].

### Virulence in *P. brasiliense*

One of our aims was to identify functional markers that could be used for detection purposes by comparing the genomes of virulent and avirulent strains. We therefore exploited the flexibility of the graph database in PanTools to link different levels of annotation while retaining all information. This allowed us to link the phenotype (virulence) with annotations such as homology groups, GO annotation or Pfam domains. Avirulent isolates appeared to be scattered throughout the phylogenetic tree; in contrast, all virulent *P. brasiliense* strains form a coherent group of highly similar genomes or a clonal lineage. Finally, two avirulent strains in this lineage were found to be genetically nearly identical to the virulent isolates. To differentiate these two avirulent strains from the highly similar virulent strains, we focused on the variation of single copy genes which represent around 95% of the individual genome gene content. We found no gene that was either extra or lost nor a SNP to discriminate these two genomes. However, genomic differences that explain the different phenotypes can possibly be found by looking into looking into the non-single-copy genes or comparing the genome structures [63]. Another promising approach would be to include intergenic regions into the pangenomic analysis. These regions account for approximately 15% of a *Pectobacterium* genome and contain important regulatory elements which play a key role in transcriptional regulation [64]. In addition to genetic or structural variation that could explain the difference in virulence, epigenetic modifications are known to result in different phenotypes as well. Through epigenetic regulation, bacteria respond quickly to environmental changes [65]. DNA methylation in particular is known to play important roles in bacterial pathogenicity [66].

We adjusted our approach to identify genes specific to the clonal lineage, allowing us to identify 86 genes only present in virulent isolates. This set of genes includes several gene candidates with functions that could contribute to the pathogenicity of *P. brasiliense* strains, such as a Lysozyme inhibitor [67], a Toll/interleukin-1 receptor [68], and a siderophore transport system [69]. Moreover, GO-terms associated, transposable elements and recombination are enriched in these genes. Combined with the fact that the 86 genes were found located largely in pairs or clusters in the genomes this further indicates that these additional genes were obtained by HGT [70] and could involve consecutive steps in pathways [71]. Instead of a single gene one or more clusters could be required for a fully virulent phenotype.

## Conclusions

This study provides a comprehensive analysis of the genus *Pectobacterium*, a diverse group of plant pathogenic bacteria of great economic importance. We have generated a pangenome from high-quality genomes using the previously published software package PanTools that was further expanded by adding new functionalities specifically designed for phylogeny and phenotypic characterization. Different methods to create phylogenies were applied that, although differing in resolution, showed very similar topology except for the tree based on gene content. This study demonstrates that gene acquisition and loss are an important source of natural variation in the genus *Pectobacterium* that can differentiate even closely related strains. In addition, clusters of genes were identified that are linked to virulence on potato under field conditions. Furthermore, we found that *Pectobacterium* spp. typically lack species-specific genes that are present in all its members, but instead present themselves as new gene combinations from the shared gene pool. The multilevel pangenomic approach allowed by PanTools, fusing DNA sequence, protein content, biological function of predicted proteins, taxonomic groups, and phenotypes, is a powerful tool to study genetic diversity and evolution.

## Methods

### Collection of strains

New isolates were obtained from asymptomatic potato tubers, symptomatic potato plants, surface water, flying insects and horticultural crop. Non-symptomatic material was ground or crushed and incubated in pectate buffer [72] before spreading on single-layer Crystal Violet Pectate (CVP) plates [73]. Characteristic colonies were subcultured on CVP and Nutrient agar plates to obtain a pure isolate. Isolates were stored at − 80 °C in 15% glycol with half strength nutrient broth.

### Phenotyping *Pectobacterium* isolates

Virulence of *Pectobacterium* isolates was assessed in seed potatoes and minitubers of the variety Kondor according to Van der Wolf et al., 2017 [19]. *Pectobacterium* strains were grown on Nutrient Agar plates for 1 day at 28 °C. Suspensions of OD600 = 0.1 were prepared in 10 mM phosphate buffer pH 7.2 and diluted an extra 100x, resulting in suspensions with about 106 CFU/ml. Potato tubers were submerged in the solution and brought under − 0.07 Pa vacuum. Vacuum was kept for 10 min, after which tubers were left submerged for another 15 min. Tubers were left to air-dry before planting. Virulence was scored as the development of typical plant symptoms [19].

### Genome sequencing

For the purpose of genome sequencing, DNA was extracted from pure bacterial cultures obtained by growing the strains on TSA medium. Per isolate approximately one inoculation loop (10 μl) of bacterial slime was collected from multiple colonies. The DNA extraction for Illumina sequencing was performed by using the Wizard® Magnetic DNA Purification System for Food (Promega, Leiden, the Netherlands) according to the manufacturer’s protocol. DNA for PacBio sequencing was obtained with the Gentra Puregene Yeast/Bact. Kit (Qiagen, Hilden, Germany) following the manufacturer’s protocol. Quantification was done by measuring the samples with the Qubit Fluorometer, using the Qubit dsDNA HS Assay Kit (ThermoFisher Scientific, Waltham, USA). DNA samples were sequenced using short read sequencing (Illumina, San Diego, USA) and long read sequencing (Pacific BioSciences, Menlo Park, USA). For Illumina sequencing a random sheared shotgun library preparation was performed using the Truseq Nano DNA Library Prep kit (dual indexing) following the manufacturer’s protocol. The samples were loaded on a paired-end flowcell, using the Hiseq PE cluster kit V4. A cBot (Illumina). One hundred twenty-five bp paired-end sequences were generated on either a Hiseq 2500 or NovaSeq 6000 device (Illumina, San Diego, USA). For PacBio sequencing DNA was sheared to 6 Kb (gTubes, Covaris) and pooled. SMRTbell™ libraries were prepared using PacBio® Barcoded Adapters for Multiplex SMRT® Sequencing according to the instructions of the manufacturer.

### De novo assembly, annotation and validation

The genomic sequences of 63 *Pectobacterium* genomes were assembled using the CLC Genomics Workbench 11.0 (https://qiagenbioinformatics.com) or Canu version 1.6 [74]**.** Publicly available *Pectobacterium* genomes were downloaded from NCBI Genbank [75] in July 2019. Genome assemblies were annotated using the Prokka pipeline (version 1.14) [32]. Very short contig sequences (< 500 bp) without gene annotation were removed as these were most likely assembly artifacts. Gene space completeness was estimated with BUSCO v3 [33] using the *Enterobacteriales* odb9 (781 orthologs) database. Genomes were removed from the dataset if the annotation completeness was below 99%, resulting in a set of 197 genomes (Additional file 1, Table S1). Biosynthetic gene clusters were predicted using antiSMASH v4.2 [76]. Functional domains and sites in protein sequences were annotated by InterProScan-5.28-67.0) [77]. Phobius [78] was used to predict transmembrane domains and signal peptides. In addition, sequences were searched against the Clusters of Orthologous Groups of proteins (COG) database [79] using BLASTP. Proteins with a hit over 65% identity and E-value below 10–5 were assigned to one of 26 COG categories [80].

### Pangenome construction and annotation

A pangenome was constructed from 197 high-quality genomes by using a modified version of PanTools [31] using a k-mer size of 17 and Prokka annotations were added. Protein sequences were clustered into homology groups using the built-in ‘group’ functionality [81]. The Gene Ontology (GO) version June 2019 (http://geneontology.org), InterPro v74 (https://ebi.ac.uk/interpro), the Pfam v32 protein family database (http://pfam.xfam.org) and TIGRFAM release 15 (https://jcvi.org/tigrfams) were integrated into the pangenome graph. Using the output of InterProScan and Phobius, gene nodes were annotated and linked to their corresponding functional annotation. Species names as well as the virulence scores were included into the pangenome as phenotypes. For the virulence phenotype, 40 *P. brasiliense* genomes were labeled as virulent or avirulent and the remaining genomes were set to unknown.

### Optimizing protein clustering and BUSCO benchmarking

The novel ‘optimal_grouping’ function of PanTools clustered the protein content eight times independently using different settings, and selected a grouping based on correct placement using 670 ‘complete’ and ‘non-duplicated’ *Enterobacteriaceae* BUSCO genes in single-copy orthology groups. Assuming each BUSCO is truly single copy, the optimal clustering setting should place each BUSCO gene in a separate homology group with one representative gene per genome. For each BUSCO gene, we checked whether its 197 members clustered into a single or into multiple groups and if they clustered with non BUSCO genes. The number of true positives (tp) and false negatives (fn) was fixed, where the highest number of correctly clustered BUSCOs present in one group are considered tp’s and the remaining BUSCO genes outside this best group are considered fn. Any other gene clustered inside BUSCO groups is considered a false positive (fp). The sums of *tp’*s, *fp’*s and *fn’*s are defined as TP, FP and FN, respectively. We measured the accuracy of each grouping by calculating recall as TP/(TP + FN), precision as TP/(TP + FP) and F-score as 2x(RecallxPrecision)/(Recall+Precision) [31].

### Establishing the core, accessory and unique part of the genome

K-mers, homology groups, and associated annotations were classified as core (present in all genomes), accessory (present in two or more genomes but not all) or unique (present in a single strain). Additional copies of a gene were considered as the same class. We classified associated annotations, such as phenotype, as specific when present in all phenotype members but absent in other genomes. Hypergeometric tests with Benjamini-Hochberg (BH) multiple testing correction [82], implemented in PanTools, were carried out on virulence associated genes to identify over-represented GO-terms. In addition, we classified k-mers and functional annotations similarly by counting their occurrence in the graph. The abundances of COG categories in a class (core, accessory, unique) were calculated and compared to obtain a fold-difference in relative abundance. We considered a COG category to be enriched for a class when the log_2_ (relative abundance) was at least 1.0 higher than the abundance of the other class.

### Determining the openness of the pangenome

Iterations of random genome combinations according to the models proposed by Tettelin et al. (2008) [36] were used to determine the contribution of new genomes with respect to the increase in core, accessory, and unique. The global gene repertoire of the *Pectobacterium* genus is represented by the total number of homology groups. To simulate the overall pangenome size increase and core genome size decrease, we iterated 10,000 times over the homology groups. Each iteration started with three random genomes from which core, accessory and unique homology groups were identified. Subsequently, random genomes were added and groups reclassified until the maximum number of genomes was reached. Heaps’ law (a power law) [36] was fitted to the number of new genes observed when increasing the pangenome by one random genome. The formula for the power law model is *n* = *k* × *N*^−α^, where n is the newly discovered genes, N is the total number of genomes, and k and α are the fitting parameters. The pangenome is open when α < 1 and closed if α > 1. To obtain the (average) increase in groups per added genome, we calculated the average number of (core, accessory and unique) groups for a certain size of the pangenome and subtracted this by the average number of groups from the pangenome with one genome less.

### Sequence alignments of homology groups

To identify variation within gene and protein sequences, nucleotide and protein sequences were extracted from homology groups and subsequently aligned with MAFFT v7.453 [49] using default settings. Edges of the initial protein alignments were trimmed up until the longest start and end gap. The original nucleotide and protein sequences were trimmed according to the protein pre-alignment and aligned a second time from which variants were extracted.

### Phylogenetic analysis

We integrated five different phylogenetic strategies into PanTools v3. All methods produce Newick formatted tree files that were visualized with iTOL [83]. To test whether the different methods were able to produce a similar phylogeny, their topologies were compared using an approximately unbiased (AU) test implemented in IQ-tree v1.6.12 [47, 48].

The first method is a MultiLocus Sequence Analysis (MLSA) that was applied to housekeeping genes: *acnA*, *dnaX*, *gyrA*, *gyrB* and *mtlD*. The initial step of the pipeline is the individual alignment of protein sequences of the five genes. Start and end gaps in the alignment were used to trim the original nucleotide sequence. A single contiguous sequence of five genes was created for each genome which were aligned by MAFFT using default settings. A maximum likelihood (ML) phylogeny was inferred from the concatenated multiple sequence alignment using IQ-tree with default settings and 1000 bootstrap iterations.

Our second method was based on single nucleotide polymorphisms (SNPs) from single-copy orthologous homology. First, single copy-groups were aligned in two consecutive rounds by MAFFT as described in the previous section. Parsimony informative positions from the nucleotide alignments were concatenated into a single contiguous sequence per genome from which IQ-tree generated a ML tree with default parameters.

For the third distance tree, Average Nucleotide Identity (ANI) scores between genomes were estimated using FastANI (version august 2019) [34]. ANI scores were transformed by 1-(ANI/100) to a distance in the range 0–1. Distances were inserted into a matrix from which we constructed a Neighbor-Joining (NJ) tree using the ape R package [84]. Furthermore, we used the ANI scores against the type strains in combination with the core SNP phylogeny to identify misclassified strains. To allow for the discovery of species-specific genes, strains were renamed when placed in a clade with different species and having an ANI score below 95% to its type strain [35, 54, 55].

The last two trees were based on distances calculated from shared gene and k-mer content. For this we recorded absence or presence of the genes or k-mers and ignored their frequency. Homology groups were utilized to identify shared genes between two genomes and k-mer sequences were counted directly in the pangenome graph. The gene distance between two genomes was obtained by calculating the Jaccard index, dividing the shared number of genes by the total number of genes. For measuring the k-mer distance, we calculated the MASH distance between two genomes as described by Ondov et al., in 2016 [85]. K-mers were disregarded when containing nucleotide codes other than the four non-ambiguous ones (A, T, C, G). Subsequently, distances were arranged into matrices from which NJ trees were inferred as described above.

## Supplementary Information

**Additional file 1: Table S1.** Genome assembly and annotation statistics of *Pectobacterium* genomes. **Table S2.** ANI scores to *Pectobacterium* type strains. **Table S3.** ANI scores of 197 *Pectobacterium* genomes. **Table S4.** Selection of 17 housekeeping genes for MLSA. **Table S5.** Pearson correlation between different phylogentic distances. **Table S6.** Number of shared and specific homology groups and functional annotations for different Pectobacterium species. **Table S7.** Avirulent specific and species-specific homology groups. **Table S8.** The genomic locations and functions of virulent associated genes in *P. brasiliense* NAK 240.

**Additional file 2: Figure S1.** Number of core, accessory and unique genes per genome. **Figure S2.** Effect of loosening core and unique threshold. **Figure S3.** Size of an 87 *P. brasiliense* pangenome. **Figure S4.** Size of a pangenome of 110 strains in 19 different *Pectobacterium* species. **Figure S5.** Size of a 27 *P. brasiliense* strain pangenome. **Figure S6.** Core SNP tree with only a single representative genome per *Pectobacterium* and *Dickeya* species. **Figure S7.** Presence of pectinase genes in 197 *Pectobacterium* genomes. **Figure S8.** Core SNP tree**. Figure S9.** Multilocus sequence analysis (MLSA) tree. **Figure S10.** Average Nucleotide Identity (ANI) tree. **Figure S11.** K-mer distance tree. **Figure S12.** Gene distance tree.

**Additional file 3.** Supplementary analyses. Functional annotation and enrichment analysis.

## Data Availability

Sequencing reads and genome assemblies are available at NCBI under project number PRJNA649220. For convenience, all genome and annotation files used for constructing the pangenome can be downloaded from 10.4121/14061122. The phylogenetic and phenotype edition of PanTools (v3) is available at: **Project name:** PanTools **Project home page:** https://git.wur.nl/bioinformatics/pantools **Operating system(s):** Unix **Programming language:** Java **Other requirements:** Required dependencies can be either installed locally or via Conda. Instructions can be found in the manual on http://www.bioinformatics.nl/pangenomics. **License:** GNU GPLv3 **Any restrictions to use by non-academics:** No

## Abbreviations

ANI: Average nucleotide identity
COG: Clusters of Orthologous Groups of proteins
FN: False negative
FP: False positive
GO: Gene Ontology
HGT: Horizontal gene transfer
ML: Maximum likelihood
MLSA: Multilocus Sequence Analysis
NJ: Neighbor-Joining
SNP: Single nucleotide polymorphism
TP: True positive
