# Supplementary material for: The Pectobacterium pangenome, with a focus on Pectobacterium brasiliense, shows a robust core and extensive exchange of genes from a shared gene pool
Source: BMC Genomics. 2021 Apr 14;22:265. doi: 10.1186/s12864-021-07583-5 (PMC8045196; doi:10.1186/s12864-021-07583-5)
Supplement: Supplementary file 2 — Additional file 2: Figure S1. Number of core, accessory and unique genes per genome. Figure S2. Effect of loosening core and unique threshold. Figure S3. Size of an 87 P. brasiliense pangenome. Figure S4. Size of a pangenome of 110 strains in 19 different Pectobacterium species. Figure S5. Size of a 27 P. brasiliense strain pangenome. Figure S6. Core SNP tree with only a single representative genome per Pectobacterium and Dickeya species. Figure S7. Presence of pectinase genes in 197 Pectobacterium genomes. Figure S8. Core SNP tree. Figure S9. Multilocus sequence analysis (MLSA) tree. Figure S10. Average Nucleotide Identity (ANI) tree. Figure S11. K-mer distance tree. Figure S12. Gene distance tree. [file 12864_2021_7583_MOESM2_ESM.pdf]

Additional file 2: Supplementary figures

The *Pectobacterium* pangenome, with a focus on *Pectobacterium brasiliense*, shows a robust core and extensive exchange of genes from a shared gene pool

Eef M. Jonkheer, Balázs Brankovics, Ilse M. Houwers, Jan M. van der Wolf, Peter J.M. Bonants, Robert A.M. Vreeburg, Robert Bollema, Jorn R. de Haan, Lidija Berke, Sandra Smit, Dick de Ridder, Theo A.J. van der Lee

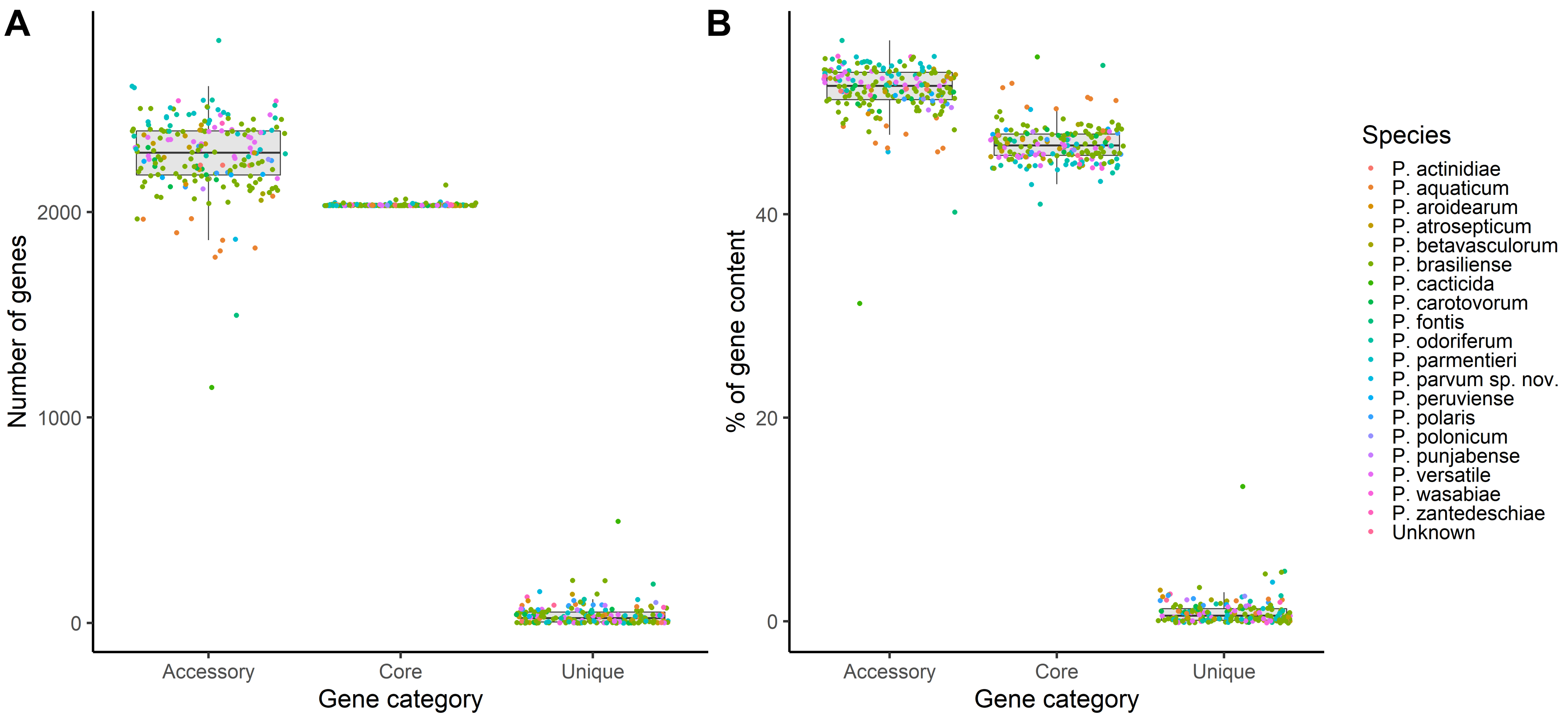

Figure S1 Number of core, accessory and unique genes per genome. The number of classified genes per genome is plotted as gene counts (a) and as percentage abundance within a genome (b).

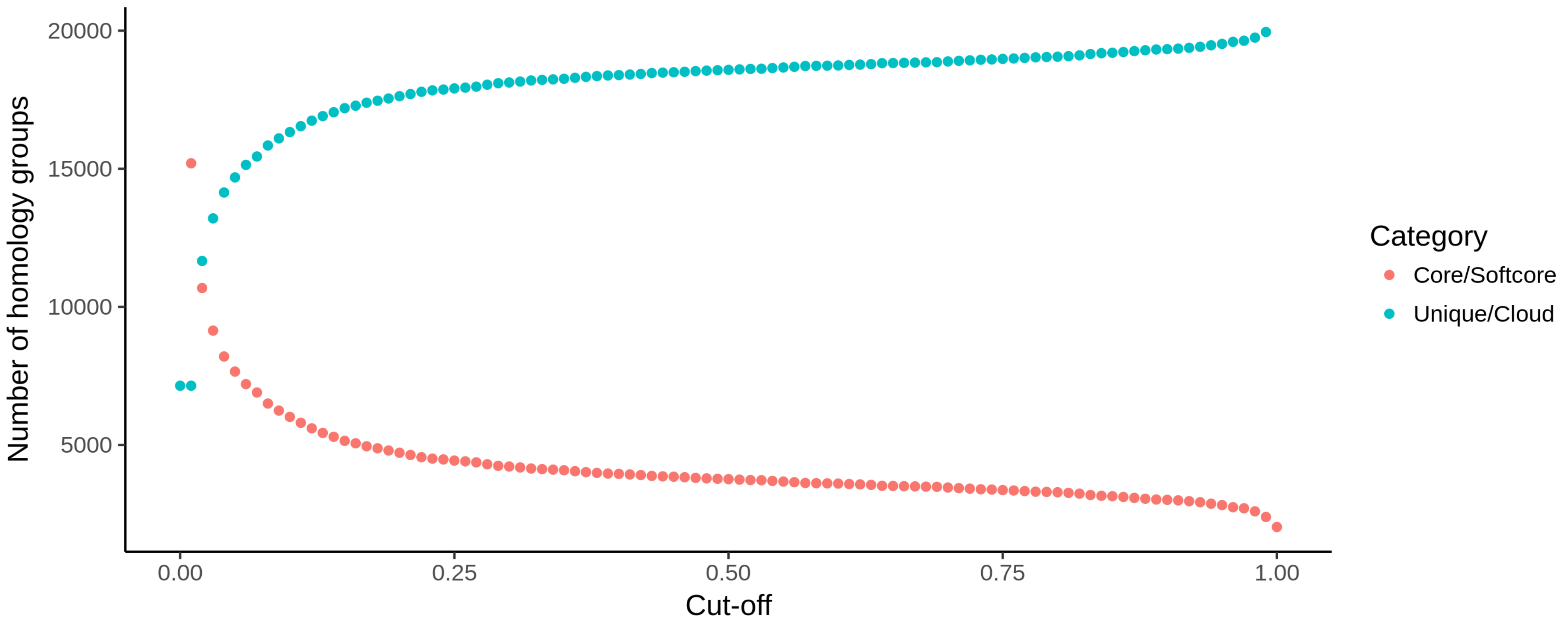

Figure S2. Effect of loosening core and unique threshold. Unique 0.00 and core 1.00 are the default thresholds of 1 and 197 genomes, respectively. Incrementing or decrementing the cut-off with one percent increases or decreases the threshold with approximately 1.97 genomes. The threshold is rounded to the nearest exact number at each cut-off.

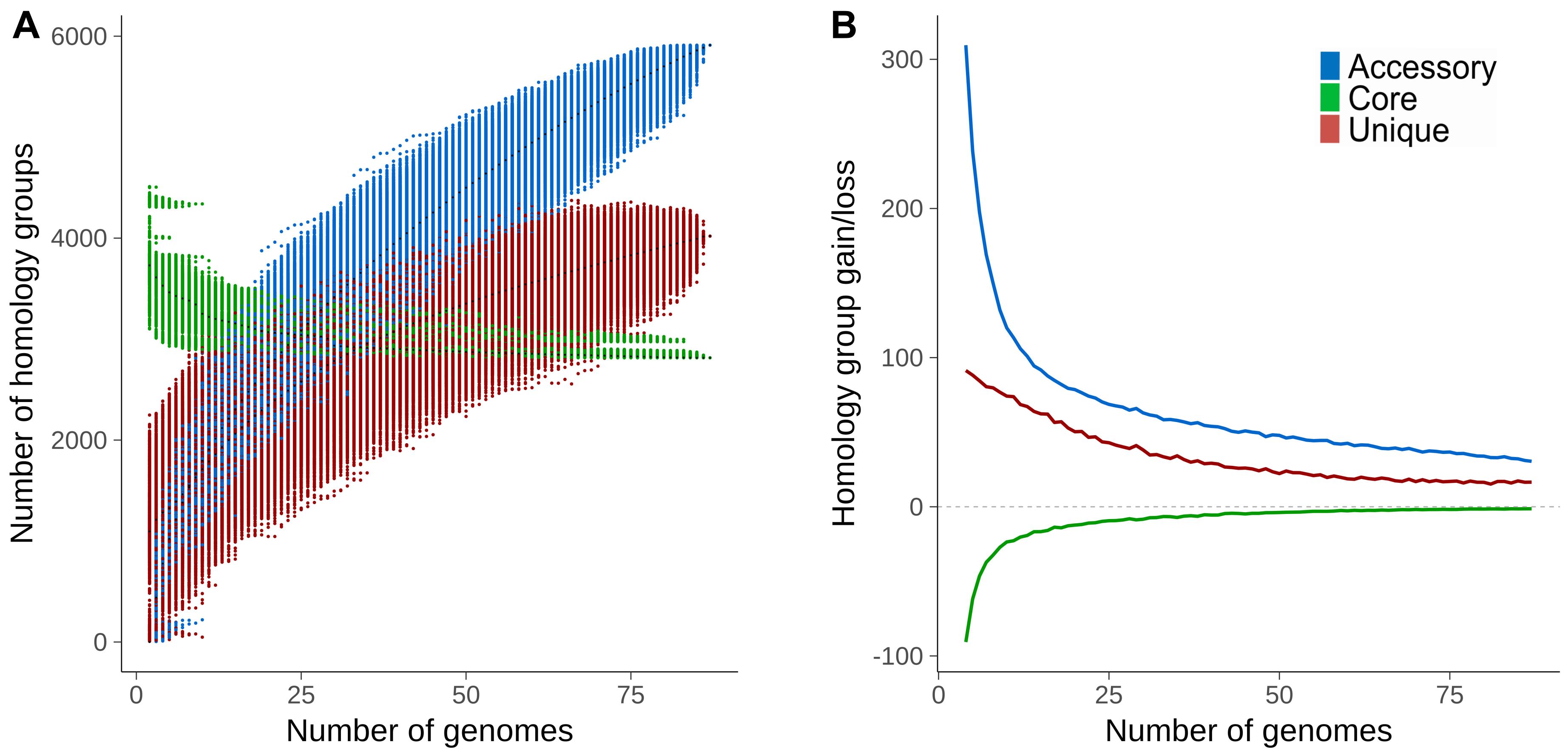

Figure S3. Size of a pangenome with 87 *P. brasiliense* strains. A. Simulation of the pangenome size calculated from 10.000 random genome combinations. **B.** The average group gain and loss by including additional genomes to the pangenome. Adding the final random genome to the pangenome, on average, lowered the number of core groups by 1.3 (σ 4.1), while the unique groups increased by 16.4 (σ 49.7), and the accessory groups increased by 29.8 (σ 32.4). The alpha value of Heaps' law is 0.51, indicating the pangenome of *P. brasiliense* is open.

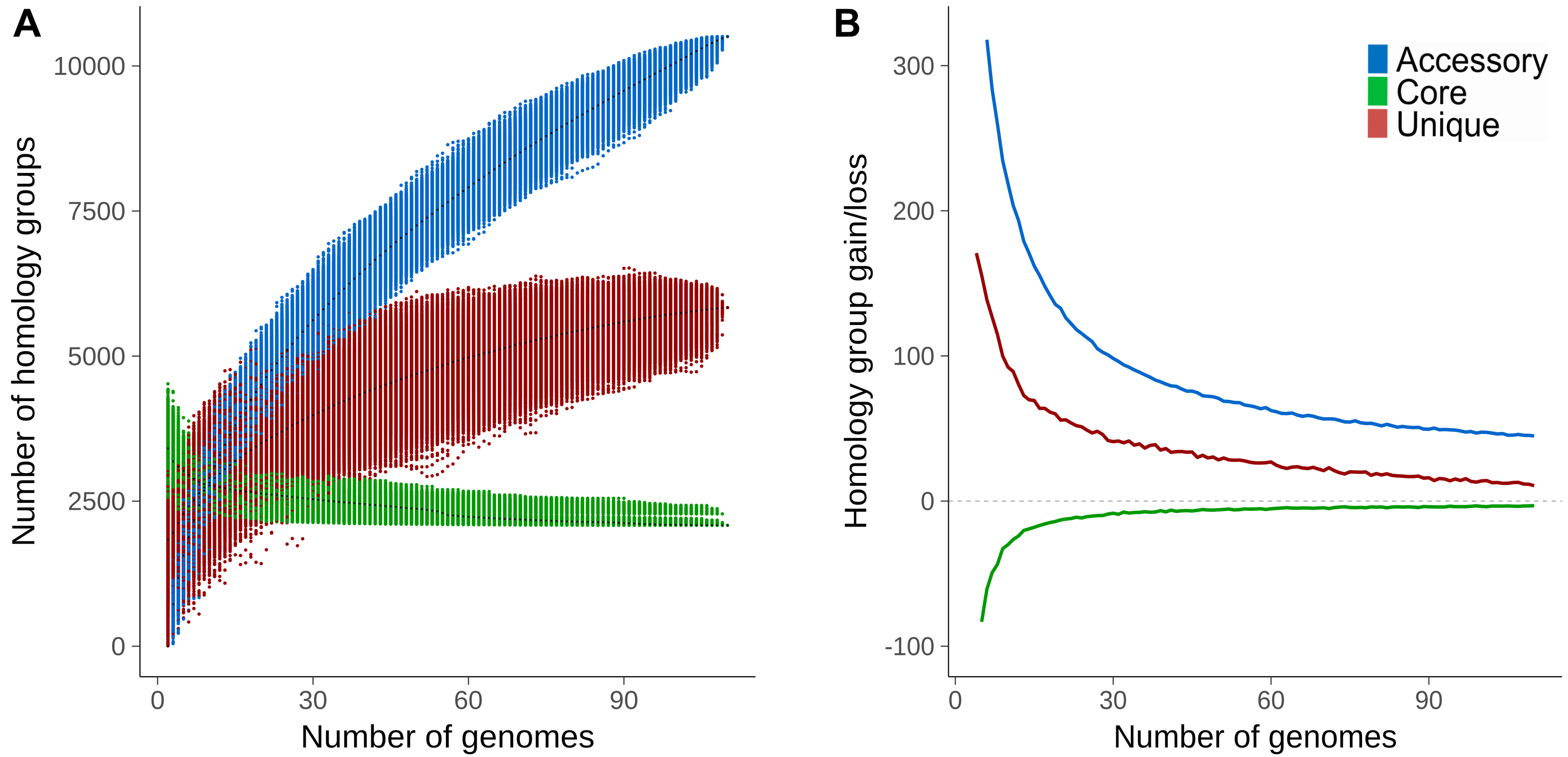

Figure S4. Size of a pangenome of 110 strains in 18 different *Pectobacterium* species. A. Simulation of the pangenome size calculated from 10.000 random genome combinations. **B.** The average group gain and loss by including additional genomes to the pangenome. Adding the final random genome to the pangenome, on average, lowered the number of core groups by 3.4 (σ 19.6), whille the unique groups increased by 11.4 (σ 70.4) and the accessory groups increased by 41.7 (σ 45.5). The alpha value of Heaps' law is 0.69, indicating the pangenome is open.

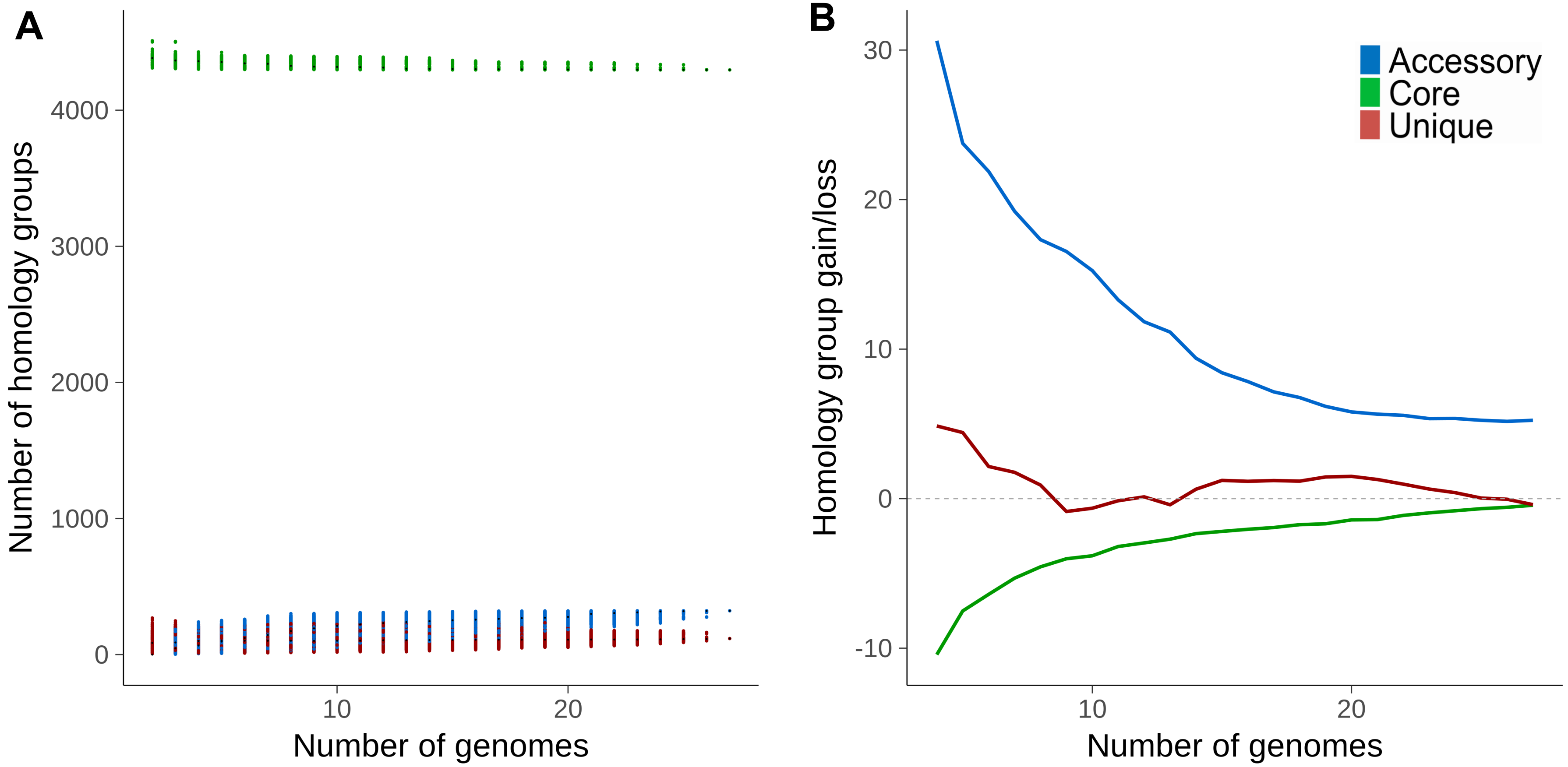

Figure S5. Size of a 27 *P. brasiliense* strain pangenome. A. Simulation of the pangenome size calculated from 10.000 random genome combinations. **B.** The average group gain and loss by including additional genomes to the pangenome. The 27 *P.brasiliense* genomes were selected based on their high similarity in the core SNP phylogeny. The alpha value of Heaps' law is 0.94, indicating the pangenome is open. Adding the final random genome to the pangenome, on average, lowered the number of core groups by 0.4 (σ 0.7), unique groups by 0.4 (σ 12.7), while the accessory groups increased by 4.7 (σ 11.9).

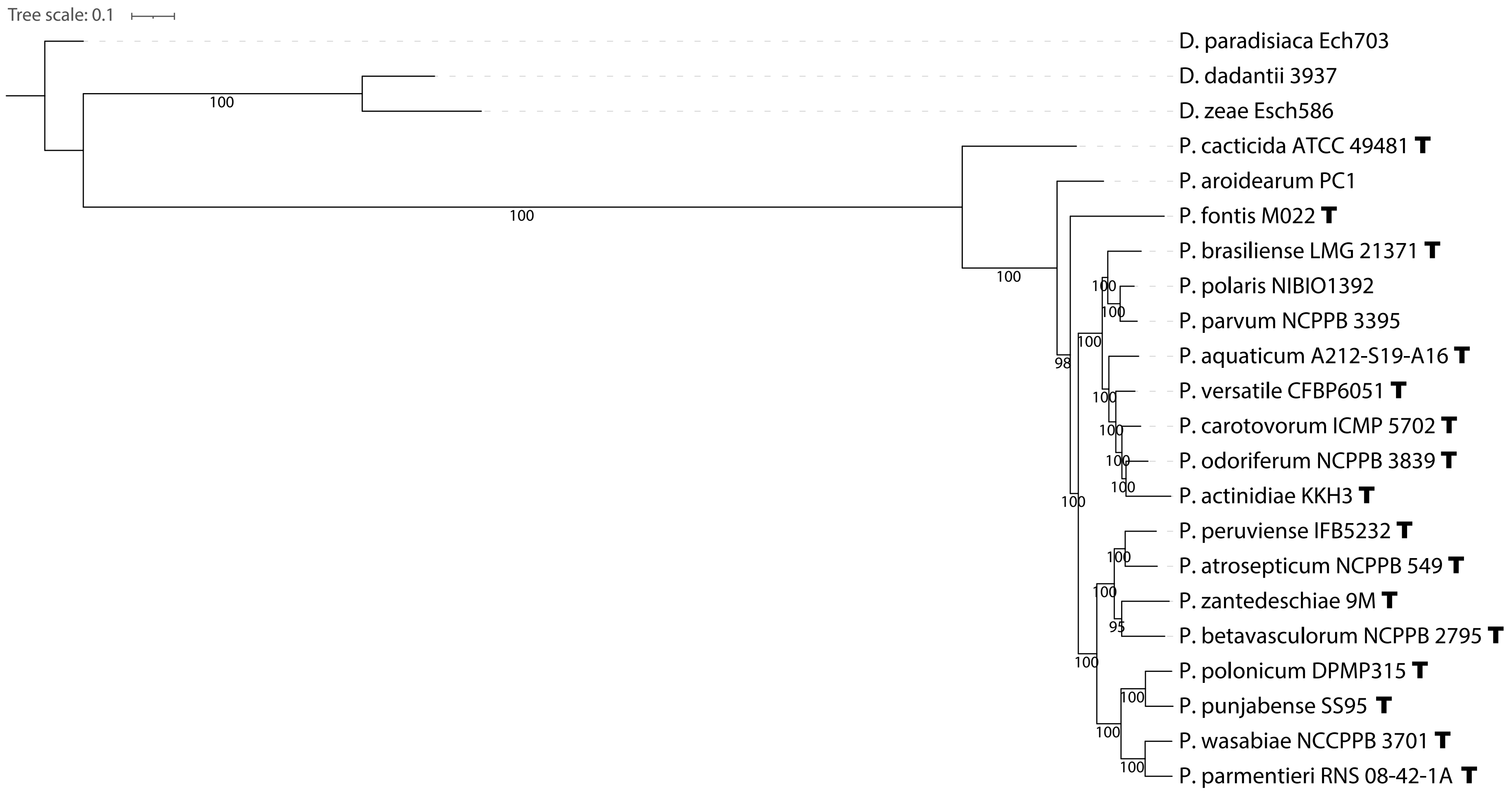

Figure S6. Core SNP tree with only a single representative genome per *Pectobacterium* and *Dickeya* species. Maximum likelihood phylogeny inferred on SNPs from 1,712 single copy-orthologous genes. The tree was rooted using *P. paradisiaca* Ech703 as outgroup. Bootstrap values from 1,000 replications are displayed.

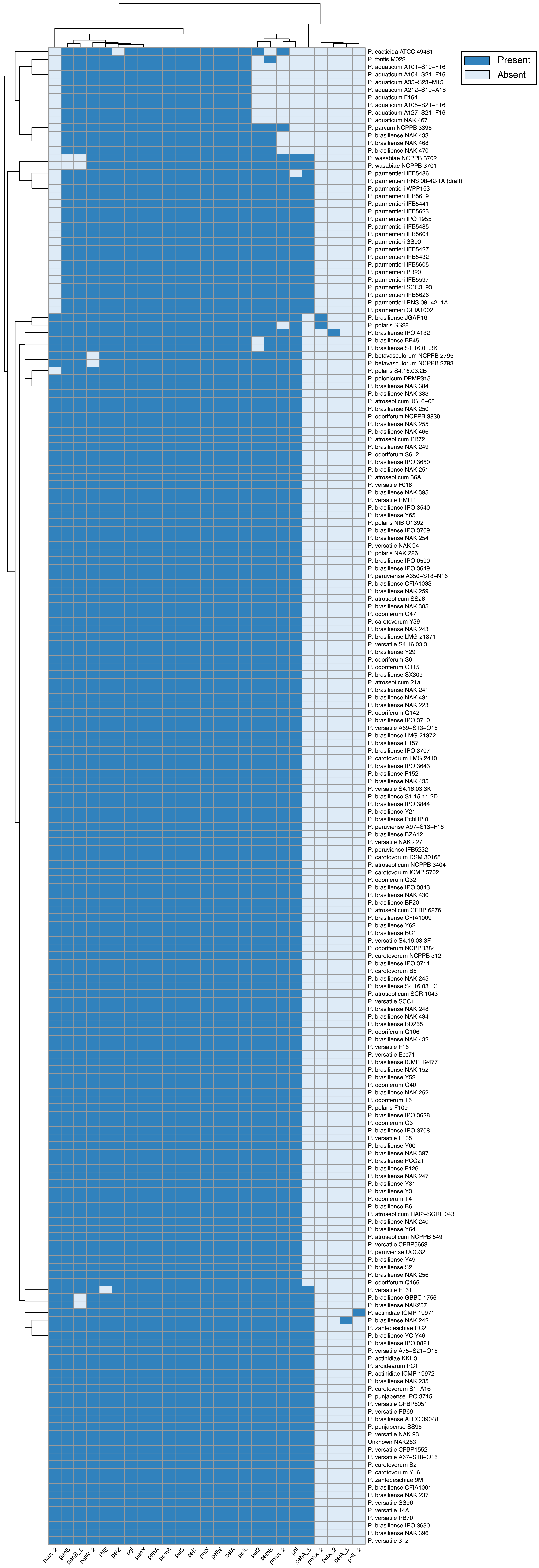

Figure S7. Presence of pectinase genes in 197 *Pectobacterium* genomes. Genes were detected through BLASTP, a hit was considered significant when it had at least 65% identity and a minimum of 65% query coverage.

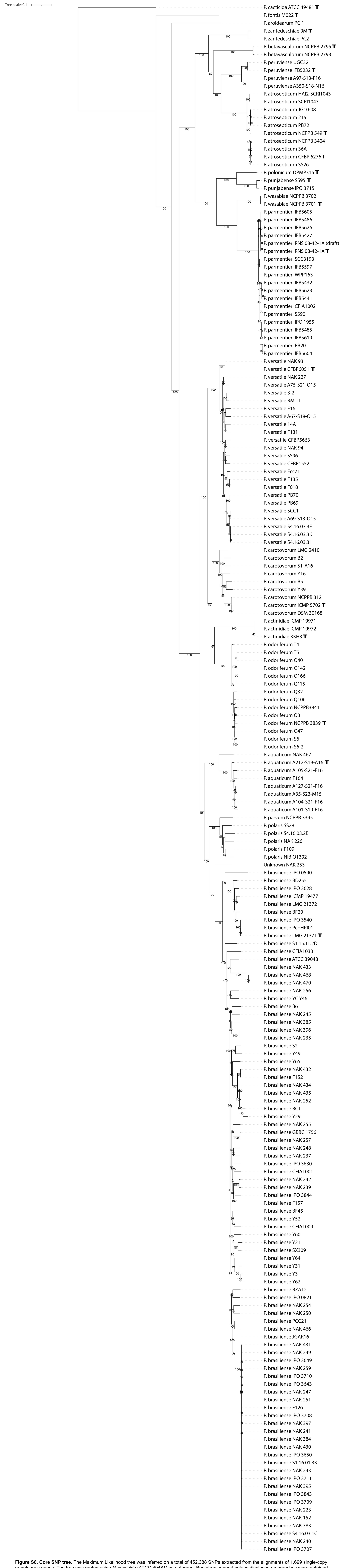

Figure S8. Core SNP tree. The Maximum Likelihood tree was inferred on a total of 452,388 SNPs extracted from the alignments of 1,699 single-copy orthologous genes. The tree was rooted using *P. cacticida* (ATCC 49481) as outgroup. Bootstrap support values displayed on branches were obtained from 1,000 bootstrap replications.

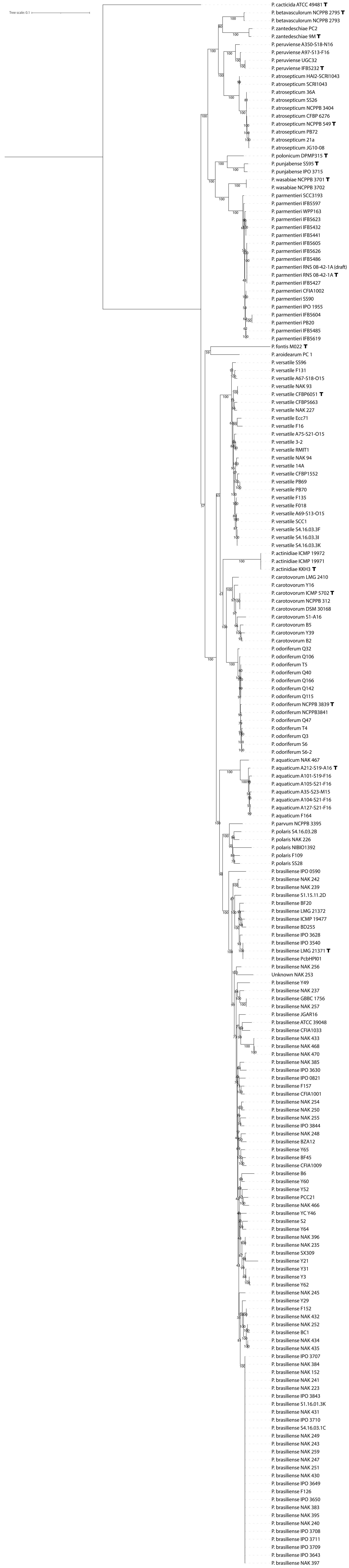

Figure S9. Multilocus sequence analysis (MLSA) tree. The Maximum Likelihood tree was inferred on the concatenated alignment of the *acnA*, *dnaX*, *gyrA*, *gyrB* and *mtlD* genes. The tree was rooted using *P. cacticida* (ATCC 49481) as outgroup. Bootstrap support values displayed on branches were obtained from 1,000 bootstrap replications.

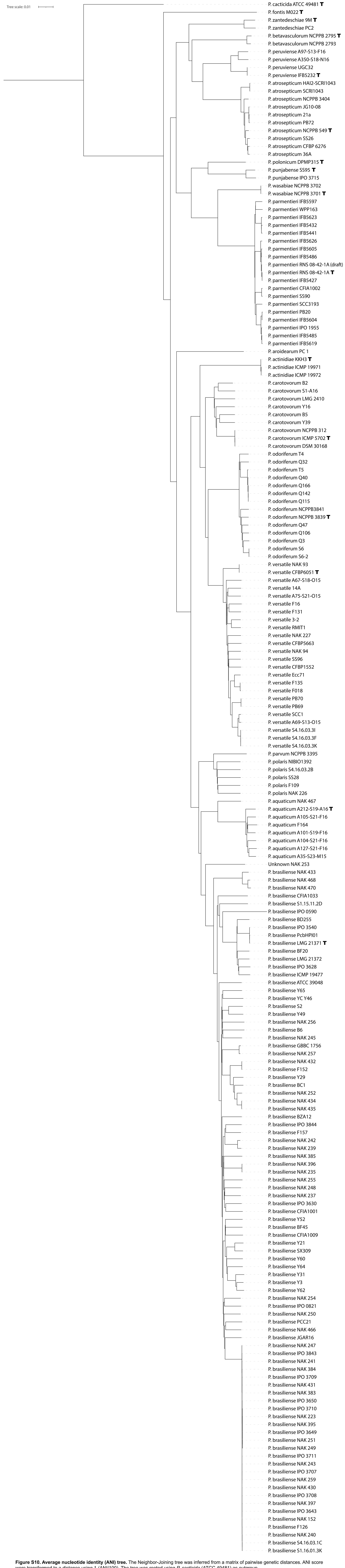

Figure S10. Average nucleotide identity (ANI) tree. The Neighbor-Joining tree was inferred from a matrix of pairwise genetic distances. ANI score were transformed to a distance using 1-(ANI/100). The tree was rooted using *P. cacticida* (ATCC 49481) as outgroup.

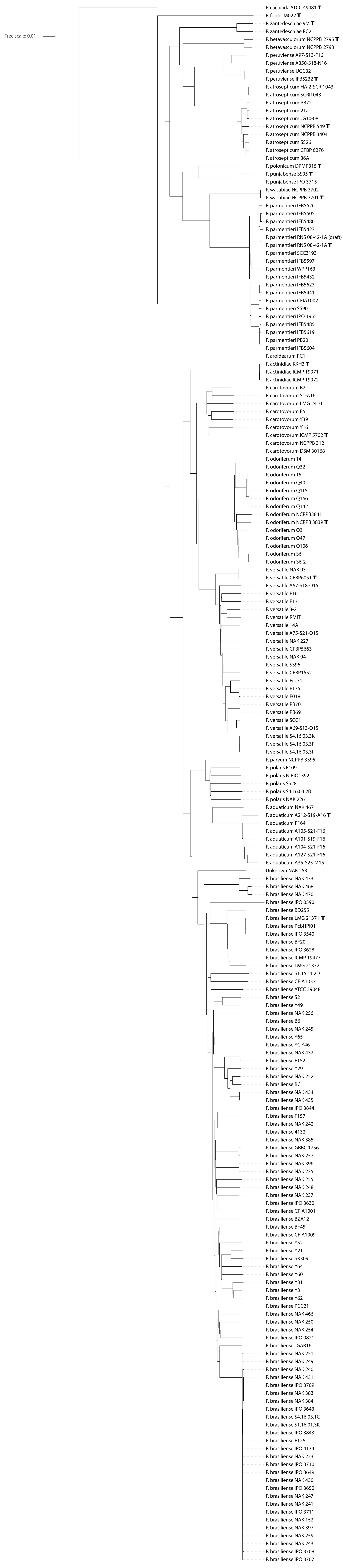

Figure S11. K-mer distance tree. The Neighbor-Joining tree was inferred from a matrix of pairwise k-mer distances. The tree was rooted using *P. cacticida* (ATCC 49481) as outgroup.

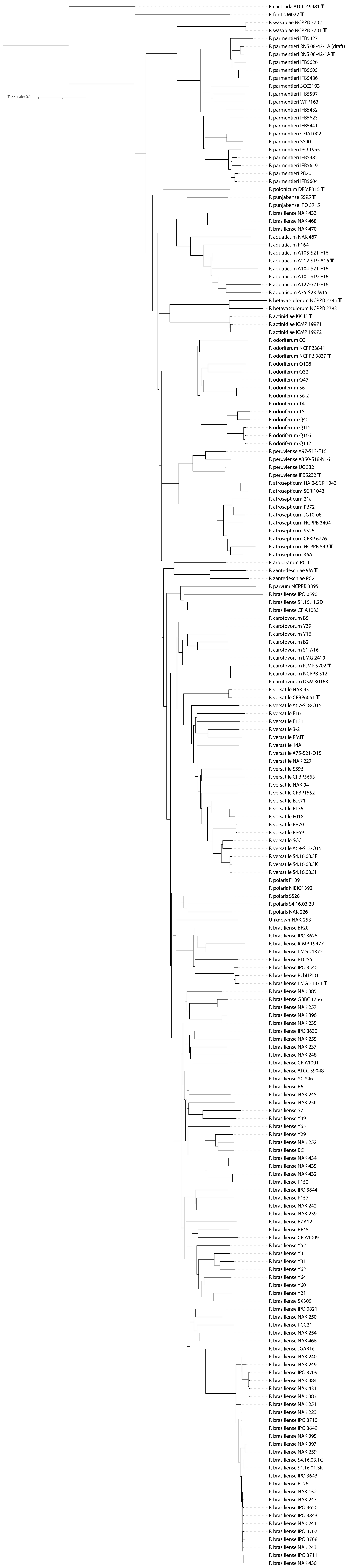

Figure S12. Gene distance tree. The Neighbor-Joining tree was inferred from a matrix of pairwise gene distances. The tree was rooted using *P. cacticida* (ATCC 49481) as outgroup.
