# Supplementary material for: The Pectobacterium pangenome, with a focus on Pectobacterium brasiliense, shows a robust core and extensive exchange of genes from a shared gene pool
Source: BMC Genomics. 2021 Apr 14;22:265. doi: 10.1186/s12864-021-07583-5 (PMC8045196; doi:10.1186/s12864-021-07583-5)
Supplement: Supplementary file 3 — Additional file 3. Supplementary analyses. Functional annotation and enrichment analysis. [file 12864_2021_7583_MOESM3_ESM.pdf]

Additional file 3: Supplementary analyses

The *Pectobacterium* pangenome, with a focus on *Pectobacterium brasiliense*, shows a robust core and extensive exchange of genes from a shared gene pool

Eef M. Jonkheer, Balázs Brankovics, Ilse M. Houwers, Jan M. van der Wolf, Peter J.M. Bonants, Robert A.M. Vreeburg, Robert Bollema, Jorn R. de Haan, Lidija Berke, Sandra Smit, Dick de Ridder, Theo A.J. van der Lee

Functional annotation and enrichment analysis

Functional annotations of virulence-specific genes

Using functional annotations integrated into the pangenome we investigated the potential functionalities of the identified virulence genes. At least one type of the following functional annotations was assigned to 55 of the 86 virulent associated homology groups: GO term, COG function, Pfam domain or signal peptide (Additional file 1: Table S8). The majority of identified functions were associated with recombination, with more than half of the proteins assigned to the COG category L: 'Replication recombination and repair' followed by X: 'Mobilome' and V: 'Defense mechanisms'. Based on the associated functions we found several genes with a putative role in virulence, such as an ABC-type siderophore export system, a lysozyme inhibitor and a Toll-interleukin-1 receptor. The genes of seven homology groups had a match to one of the databases used during the Prokka annotation and were assigned a gene name. Five of these genes are known to be involved in recombination (*bin3*, *hsdM*, *hsdS*, *intA*, *recF),* one gene (*yojI*) is an ABC transporter and one gene (*cas2*) is part of the bacterial adaptive immunity system CRISPR-Cas.

GO enrichment analysis on virulence-specific genes

GO enrichment analysis was performed on the genes with at least one GO term (16 groups) to identify functions overrepresented w.r.t. to the remainder of the genome. In total, 90 terms were connected to the groups of which 31 were enriched after Benjamini and Hochberg multiple testing correction ($p < 0.01$) (Table S8, Figure S13-S14). No difference in enriched functions was found between virulent strains, as these genomes were highly similar. As expected, we found enriched functions that were in line with the found COG categories; GO:0006310: DNA recombination and GO:0003677: DNA binding that can be explained as remnants of horizontal gene transfer and transposon activity. More interestingly, GO:0032775: DNA methylation on adenine and its parent term GO:0006306: DNA methylation were highly over-represented functions since half of these two specific GO terms were found in the virulent associated genes.

Virulent-specific functional annotations

In addition to virulent-specific genes, we also investigated whether there are differences in protein domains between the two groups. Four Pfam domains were found in all virulent strains and absent in 23 of the 25 avirulent strains. Three identified domains were part of proteins in virulent specific homology groups that we described above: PF13079: Unknown function, PF15599: Immunity protein 63 and PF01443: Viral RNA helicase. The fourth domain, PF14021: Tuberculosis necrotizing toxin, was only found in particular copies of *cdiA*. Proteins from the *cdiA* gene family varied from 1,107 to 5,809aa in length, were highly variable and therefore were clustered into four separate homology groups of which none were specific to genomes of virulent strains. All but five strains in the *Pectobacterium* genus have at least one copy of the *cdiA* gene; however, the functional domain was present in only 34 strains, including all virulent *P. brasiliense* and the avirulent NAK 223 and NAK 259 strains. Despite the fact that proteins were highly variable, the PF14021 domain was always found around hundred amino acids in front of the C-terminus.

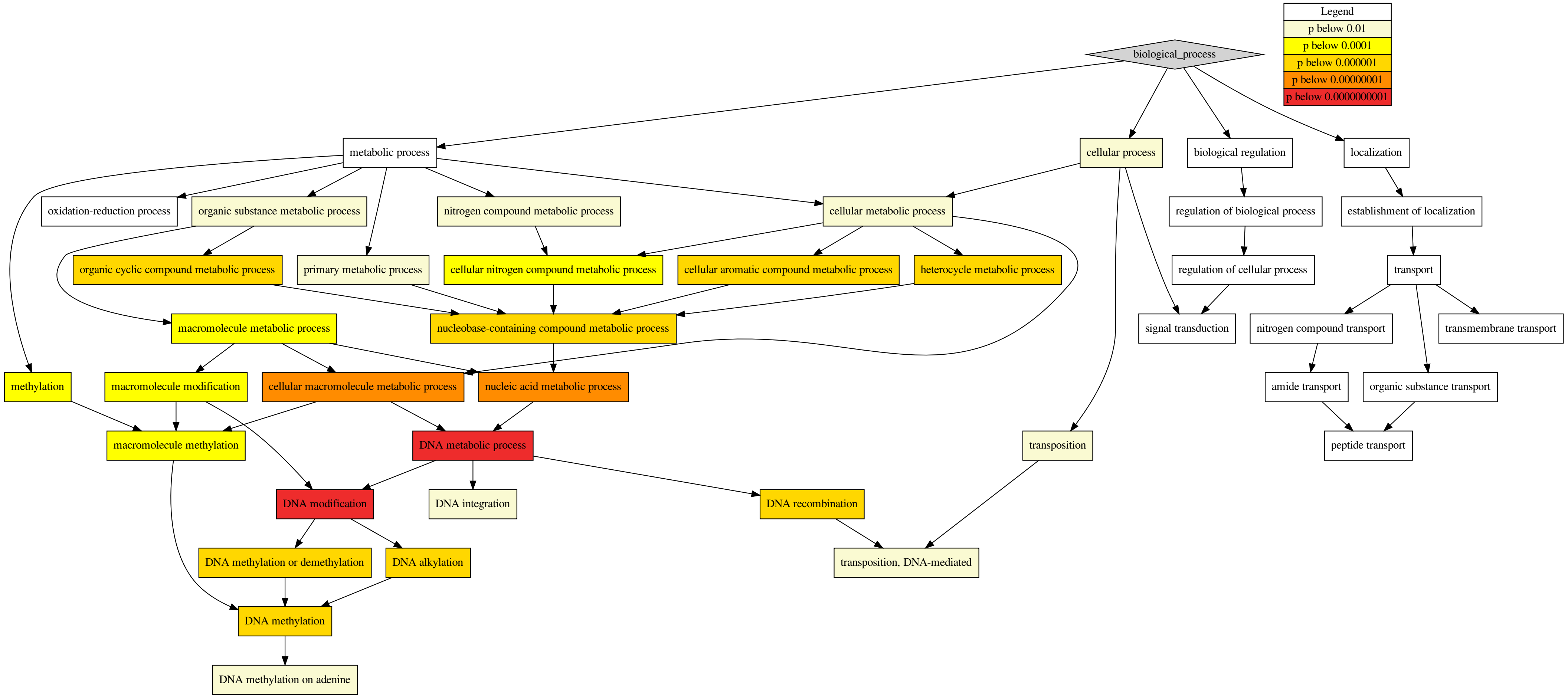

Figure S13. Biological process GO-terms of 88 virulence-associated genes in *P. brasiliense* NAK 240. The color of the GO-term indicates the p-value from the hypergeometric test. Every arrow represents an 'is_a' relationship.

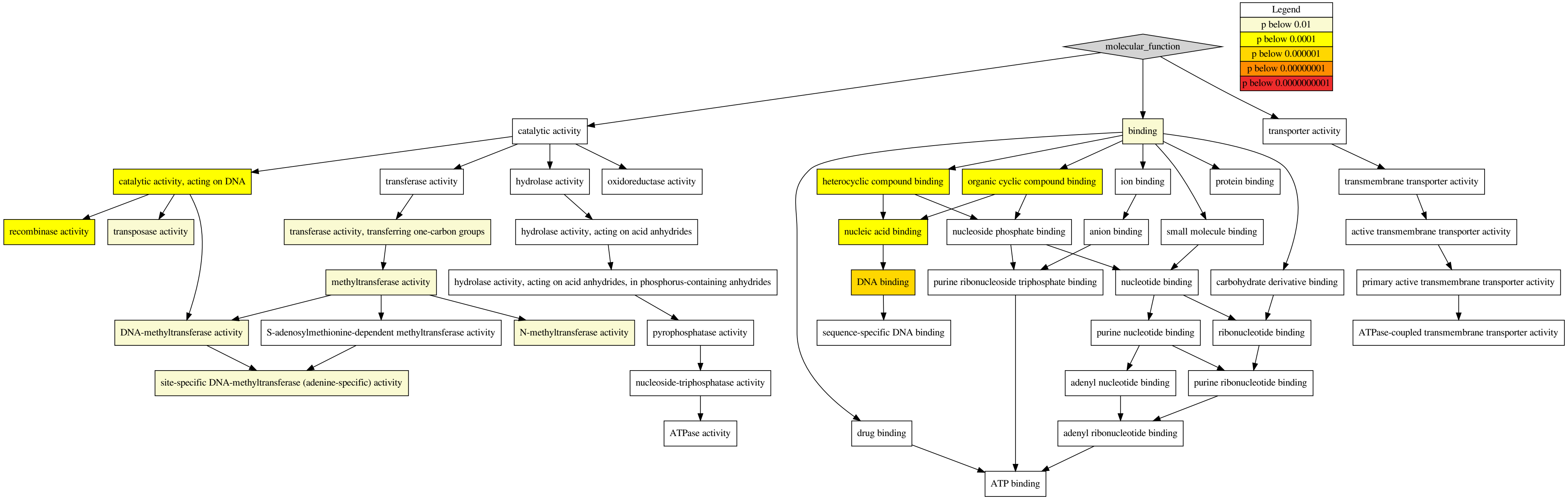

Figure S14. Molecular function GO-terms of 88 virulence-associated genes in *P. brasiliense* NAK 240. The color of the GO-term indicates the p-value from the hypergeometric test. Every arrow represents an ‘is_a’ relationship.
